# Membrane Sculpting by F-BAR Domains Studied by Molecular Dynamics Simulations

**DOI:** 10.1371/journal.pcbi.1002892

**Published:** 2013-01-31

**Authors:** Hang Yu, Klaus Schulten

**Affiliations:** 1 Beckman Institute, University of Illinois, Urbana, Illinois, United States of America; 2 Center of Biophysics and Computational Biology, University of Illinois, Urbana, Illinois, United States of America; 3 Department of Physics, University of Illinois, Urbana, Illinois, United States of America; Max Planck Institute for Biophysical Chemistry, Gottingen, Germany

## Abstract

Interplay between cellular membranes and their peripheral proteins drives many processes in eukaryotic cells. Proteins of the Bin/Amphiphysin/Rvs (BAR) domain family, in particular, play a role in cellular morphogenesis, for example curving planar membranes into tubular membranes. However, it is still unclear how F-BAR domain proteins act on membranes. Electron microscopy revealed that, *in vitro*, F-BAR proteins form regular lattices on cylindrically deformed membrane surfaces. Using all-atom and coarse-grained (CG) molecular dynamics simulations, we show that such lattices, indeed, induce tubes of observed radii. A 250 ns all-atom simulation reveals that F-BAR domain curves membranes via the so-called scaffolding mechanism. Plasticity of the F-BAR domain permits conformational change in response to membrane interaction, via partial unwinding of the domains 3-helix bundle structure. A CG simulation covering more than 350 *µs* provides a dynamic picture of membrane tubulation by lattices of F-BAR domains. A series of CG simulations identified the optimal lattice type for membrane sculpting, which matches closely the lattices seen through cryo-electron microscopy.

## Author Summary

To generate organelles, eukaryotic cells sculpt their membranes into compartments, often employing proteins as chaperones, for example, F-BAR domains. The latter induce formation of tubular and vesicular membranes. Functional and structural studies suggest that F-BAR domains sculpt membranes through electrostatic interactions, driving the membrane to match the concave surface of the protein's banana-like shape. Cryo-electron microscopy (cryo-EM) studies provide an average static picture of how F-BAR domains form lattices on the surface of membranes to induce tube formation. Complementing the cryo-EM images, molecular dynamics simulations reported here offer a detailed, dynamic picture of membrane tubulation by a lattice of F-BAR domains and identified lattice types optimally attuned to producing high membrane curvature. The simulations reproduced also a process lasting 350 µ*s* in which lattices of F-BAR domains form a complete tube out of an initially flat membrane. The molecular dynamics study offers, thereby, both a large-scale picture of membrane sculpting by F-BAR domain lattices as well as atomic-level dynamic information about the involvement of the individual F-BAR domain and its interactions with partner F-BAR domains and membrane in the sculpting process.

## Introduction

Interplay between cellular membranes and their peripheral proteins drives many cellular processes, including cell division, growth, movement and cell-cell communication [1]–[6]. During their lifetime and often with the help of membrane peripheral proteins, eukaryotic cells dynamically sculpt their various types of compartments [2], [5], [7]–[12]. Recently, increasing attention has been paid to these proteins [11]–[23].

Proteins of the Bin/Amphiphysin/Rvs (BAR) domain family play an important role in membrane remodeling, by inducing and stabilizing membrane curvature [13],[24]–[26]. For example, BAR domain deficiency is related to a wide range of cancers and blood disorders [27]. Resolved structures show that BAR domains form crescent-shaped homodimers, the monomers being composed of coiled-coil association of a 3-helix bundle structure [13], [28]–[31]. Three sub-families of BAR domains, namely N-BAR domains, FCH-BAR (F-BAR) domains and Inverse-BAR (I-BAR) domains, differ from each other in their structure and physiological function [7], [32]–[36]. In contrast to N-BAR domains that form a banana shaped dimer, F-BAR domains are elongated and only gently curved [37], [38]. A high density of positive charge is found on the part of the protein that is destined to interact with negatively-charged membranes [2], [30], [39], [40]. While N-BAR domains stabilize highly curved membrane structures, F-BAR domains stabilize membrane structures of small degree of curvature [13], [30], [32], [38], [41]. N-BAR domains also have an N-terminal amphipathic helix, which aids membrane curvature stabilization by membrane insertion. Such helix is lacking in the case of F-BAR domains [37], [38]. Both N-BAR domains and F-BAR domains are found to induce formation of tubules *in vitro* [17], [18], [37].

Two mechanisms of membrane curvature generation by BAR domain proteins have been proposed [7], [13], [35], [36], [38]. According to the scaffolding mechanism, BAR domains bend membranes by attracting negatively-charged lipid headgroups to their positively-charged curved surface [7], [13], [36], [38]. During the scaffolding process, a BAR domain is considered to act as a rigid body, to which lipids are attracted via electrostatic interaction, transferring membrane binding energy into membrane bending energy [36], [42]. According to the membrane insertion mechanism, a BAR domain inserts its amphipathic groups like wedges into one leaflet of the membrane and, thereby, curves the membrane [2], [35], [38]. N-BAR proteins use their N-helix as an amphipathic wedge, while for the F-BAR domain it is suspected that residue Phe117 inserts its bulky side chain into the membrane [7], [30], [38], [43]–[46]. Either mechanism involves strong membrane-protein interactions.

BAR domains are found to shape low-curvature liposomes into high-curvature tubules *in vitro* [7], [38], [47]. Such extensive membrane remodeling requires collective action of multiple BAR domains. Striations observed on the surface of BAR domain-induced tubules suggest that the tubules are covered by an ordered arrangement of the proteins [7], [19], [38], [47]. Recent observations revealed that well-organized spirals of BAR domains form on the surface of membrane tubules [19], [38]. Differences in lattices formed by BAR domains may result in variations of membrane curvature and structure [38]. However, it remains unclear how membrane curvature depends on the type of F-BAR domain lattice arrangement. Two further open questions are: How do individual F-BAR domains interact with a membrane to form local curvature? What dynamics is involved in membrane curvature formation by F-BAR domain lattices?

Computational approaches, especially molecular dynamics (MD) simulation, are proven tools for the study of membrane-protein interactions [48]–[58]. Recent studies on membrane deformation by BAR domain proteins include the study of local deformation of membranes by single N-BAR domains [12], [33], [46], [59], of large-scale membrane structure deformation by multiple N-BAR domains described by coarse-grained models [24], [25], [60]–[62] and of large scale membrane deformation under the influence of BAR domains [63]–[66].

Extending previous studies [24], [25], [60], [61], we present here the first all-atom molecular dynamics simulations of F-BAR domains acting on a lipid bilayer in a fully solvated system. We explore the system in an equilibrated state without restraints and seek to reveal how F-BAR domains produce membrane curvature by conformational change of their coiled-coil 3-helix bundle structure. We also test the mechanism underlying membrane bending by mutating key positively-charged residues of the F-BAR domain. We then employ a shape-based coarse-grained (SBCG) model developed in our group [60] to examine the effect of the F-BAR domain lattice arrangement on membrane sculpting; variations of the lattice are found to form a wide range of membrane curvatures. Finally, we demonstrate through simulations how F-BAR domain lattices form a complete membrane tubule.

## Results/Discussion

F-BAR domains are known to bind to membrane surfaces and generate membrane vesicles as well as tubules with radii in the range 25–100 nm [7], [19], [38], [47], [67]. To reveal the mechanism of membrane curvature generation by F-BAR domains, we employ all-atom and coarse-grained molecular dynamics simulations to characterize the effect of F-BAR domains on membrane curvature. Table 1 lists size and timescale of the simulations carried out and demonstrates the multiscale nature of the present study.

**Table 1:** Simulations performed in this study.

Simulation	Description	Time	Type	Size (nm)	Atoms/Beads
WT1	Wild type, equilibration 1 of a single wild type F-BAR domain on top of a lipid patch (Fig. 9)	250 ns	all-atom	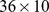	0.4 M
WT2	Wild type, equilibration 2 of a single wild type F-BAR domain on top of a lipid patch (Fig. 9)	175 ns	all-atom	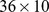	0.4 M
NC	No charge, equilibration of a single F-BAR domain with positive charges on certain residues abolished, on top of a lipid patch	80 ns	all-atom	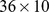	0.4 M
NL1	No lipid, equilibration 1 of a single wild type F-BAR domain in water	160 ns	all-atom	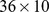	0.4 M
NL2	No lipid, equilibration 2 of single wild type F-BAR domain in water	160 ns	all-atom	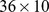	0.4 M
WT1DEL	Starting from final frame of WT1, residues 56 to 60 of the F-BAR domain are deleted	25 ns	all-atom	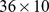	0.4 M
WT1WAT	Starting from final frame of WT1, the membrane is removed and the F-BAR domain is solvated in water	85 ns	all-atom	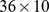	0.4 M
8 F-BARs	Equilibration of 8 wild type F-BAR domains on top of a lipid patch (Fig. S11 in Text S1)	175 ns	all-atom	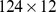	3.6 M
LATTICES	More than 30 simulations with different F-BAR domain lattices on top of a lipid patch (Fig. 4)	3 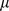 s	SBCG	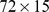	3666
SBCG 8 F-BARs	Equilibration of 8 SBCG F-BAR domains on top of a lipid patch (Fig. S11 in Text S1).	175 ns	SBCG	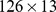	3000
TUBULATION	68 F-BAR domains arranged in a lattice on top of a large lipid patch (Fig. 6, 7 and Video S3)	350 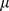 s	SBCG	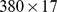	21800

### The F-BAR domain binds and curves a membrane via scaffolding

**Figure 1:**
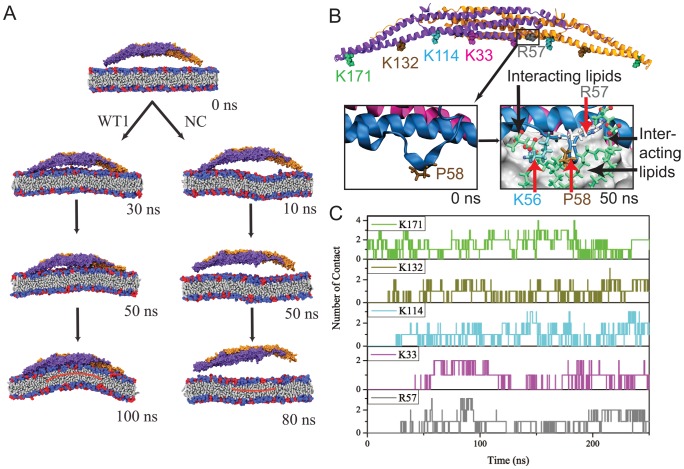
Interaction of an individual F-BAR domain with a lipid membrane. (A) Lipid membrane interaction with the wild type F-BAR (WT) domain (as described in simulation WT1) and F-BAR domain with positive charges on residues along the inner leaflet abolished (as described in Methods for simulation NC). WT binds to the membrane in 30 ns and generates a 28 nm radius of curvature within 100 ns. In the case of NC, the F-BAR domain does not bind to the membrane over 80 ns and the membrane remains flat. Membrane lipids are colored in grey; F-BAR proteins are colored in blue and orange to distinguish the monomers. (B) Locations of residues 56 to 60 and the positively-charged residues along the inner surface of the F-BAR dimer. Location of residues 56 to 60 at time 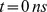 (insert left) and 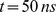 (insert right); the membrane is shown in grey surface representation; F-BAR proteins are colored in blue and orange to distinguish the monomers. Representative residues interacting with lipid are colored in green, brown, blue, purple and grey as well as highlighted by red arrows; interacting lipids are shown in green stick representation. (C) Number of contacts formed between negatively-charged DOPS lipid headgroups and positively charged residues along the inner surface of F-BAR domains. A contact is considered formed if nitrogen atoms of Arg/Lys residues are within 5*A?* of an oxygen atom of a DOPS lipid headgroup. Contact of representative residues with lipid are colored in green, brown, blue, purple and grey as in (B). Additional contacting residues are shown in Fig. S1 in Text S1.

The results of two separate all-atom equilibrium simulations of single F-BAR domains (WT1 and NC) binding to negatively charged lipid bilayers, consisting of 33% DOPS and 67% DOPC, are shown in Fig. 1A and Videos S1 and S2. An F-BAR domain dimer was placed on top of the resulting patch with no initial contacts to the membrane. In simulation WT1, the wild type F-BAR domain was employed; in simulation NC, the positive charges of selected residues (see Methods) along the inner surface of the wild type F-BAR domain were neutralized without changing residue structure.

In simulation WT1, the wild type F-BAR domain binds to the membrane within 30 ns, at which moment most positively charged residues are in close contact with the negative charges on DOPS headgroups (Fig. 1B); at this point the membrane curvature gradually increases to reach a maximum within 100 ns. Several positively charged residues are found to form close contacts with negatively charged DOPS headgroups. Two clusters of positively charged residues, cluster 1 (residues Lys27, Lys30, Lys33, Lys110, Arg113, Lys114, Arg121, Arg122) located at the center of the F-BAR domain and represented by Lys114 and Lys33, and cluster 2 (residues Lys132, Arg139, Lys140, Arg146, Lys150) represented by Lys132 and located at the side helices of the F-BAR domain, are found to form extensive contacts with DOPS headgroups in the course of the simulation (Fig. 1C and Fig. S1 in Text S1). Indeed, clusters 1 and 2 are important for binding and membrane curvature formation; mutation of the residues mentioned can abolish lattice formation [38]; most of the stated residues are conserved in both their sequence and structural context across different species and different F-BAR domains (Fig. S2 and Fig. S3 in Text S1). In contrast, residues Lys138 and Lys173 do not form contacts with the negatively charged membrane, suggesting that their main function is to form salt bridges with neighboring residues to maintain the F-BAR domain structure (Fig. S1 in Text S1).

Several positively charged residues, namely arginine residues Arg27, Arg113 and Arg121, are also found to interact with lipids while at the same time interacting with negative charges on the F-BAR domains (Fig. S1 and Fig. S4 in Text S1), suggesting that these positively charged residues play both structural and membrane binding/bending roles. The arginine residues interact with neighboring negatively charged residues to maintain the F-BAR domain structure, while interacting with lipids to anchor the F-BAR domain to the membrane. Residue Phe117 had been suggested to induce membrane deformation by membrane insertion [30], [38]. However, over the course of simulations WT1 and WT2, residue Phe117 is found to get buried inside the protein helix bundle and not to form contacts with the membrane (Fig. S1B in Text S1).

Binding of the wild type F-BAR domain to the membrane occurs sequentially, from sides to center (Fig. 1C). Contacts between residue cluster 1 (represented by residue Lys171) and negatively charged lipids forms within the first few nanoseconds of simulations WT1, showing that cluster 1 residues play a key role in adhering the protein to the membrane. Contacts between cluster 2 (represented by residue Lys132) and membrane form last, suggesting that cluster 2 residues are important for curvature generation, by attracting lipid to the protein. All contacts between positively charged residues and membrane formed within 40 ns of simulation WT1.

### Side loops formed by residues 56 to 60 maintain the F-BAR domain in an upright orientation

As shown in Fig. 1C, residue Arg57 forms a long lasting contact with the membrane. This residue is located on a short loop formed by residues 56 to 60. This loop contains dense positive charges (Lys56, Arg57, Lys60) and partially inserts Pro58 into the membrane. However, the insertion did not occur until 80 ns in simulation WT1, i.e., after the protein is fully bound to the membrane. The absence of loop insertion during the early stage of protein-membrane interaction suggests that the 56–60 loop does not contribute directly to membrane binding or initial curvature development. The area of the membrane taken up by the loop is 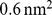, which is much smaller than the membrane area taken by the N-helix of the N-BAR domain. According to [63], to effectively deform a membrane of 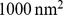 with loop insertion, at least 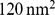 membrane area needs to be taken up by the protein insertions, corresponding to 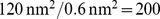 loops, i.e., 100 F-BAR dimers. However, the area of membrane plane taken by an F-BAR domain is 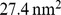 per dimer and for 100 F-BAR dimers, a lipid area of 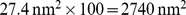 is required. It is impossible to place 100 F-BAR dimers onto a 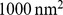 membrane in an orientation that both loops of each dimer contact the membrane. Therefore, it is unlikely that the loop is involved in a major way in membrane bending. Indeed, removing residues 56 to 60 showed no significant change in membrane curvature during a 40 ns simulation (WT1DEL, see Table 1), strengthening further the conclusion that membrane insertion by the short loop does not contribute significantly to membrane curvature formation (Fig. S5 in Text S1). However, the F-BAR domain turning from an upright orientation to a side-laying orientation was observed from 40 ns onwards and the membrane curvature was found to decrease at the same time (Fig. S5 in Text S1). In experiments, side-laying states are observed at low BAR domain density and induce tubules of low curvatures [9], [38]. Therefore, the function of the 56–60 loop is likely a structural one, namely maintaining the F-BAR domain in an upright orientation and forming contacts with the membrane; the function of the F-BAR domain loop is similar to the function of N-helices in case of N-BAR domains.

### The F-BAR domain undergoes conformational change during membrane curvature generation

**Figure 2:**
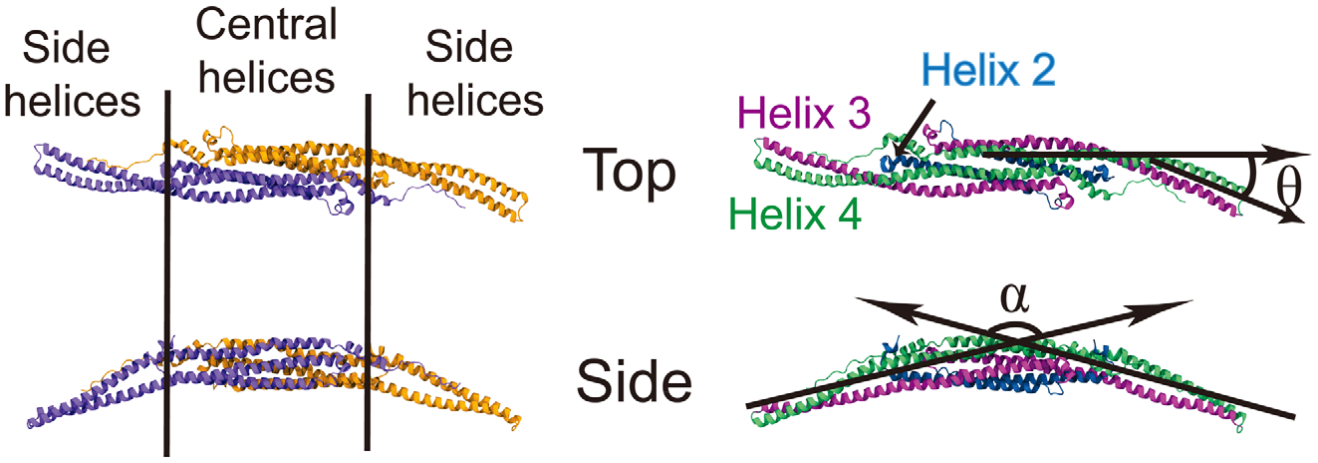
Conformation of F-BAR domain characterized through angles 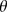
 **and** 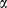 **.** (Left) The F-BAR domain is a dimer of two F-BAR proteins, the latter colored in purple and orange. (Right) Each F-BAR protein is composed of five helices forming a coiled-coil structure, with helix 2 to 4 colored blue, purple and green, respectively. 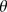 is formed by the principal axes of the central part and the end part of helix 4 (green); 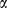 is formed by the angle between the principal axes of the left and the right sides of helix 3 (purple).

During the process of curvature generation, the F-BAR domain interacts with the membrane and undergoes a large conformational change involving its side helices (helices 3 and 4, see Fig. 2). To represent the change we employ angle 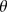 and 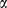. 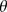 is formed by the principal axes of the central helix 4 (green, residues 241 to 257) and side helix 4 (green, residues 182 to 204); a decrease of the 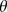 value corresponds to a straightening of the domain. 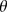 is formed by the angle between the principal axes of the left and right sides of helix 3 (purple, residue 120 to 166); a decrease of the 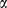 value corresponds to an increase of overall domain curvature.

**Figure 3:**
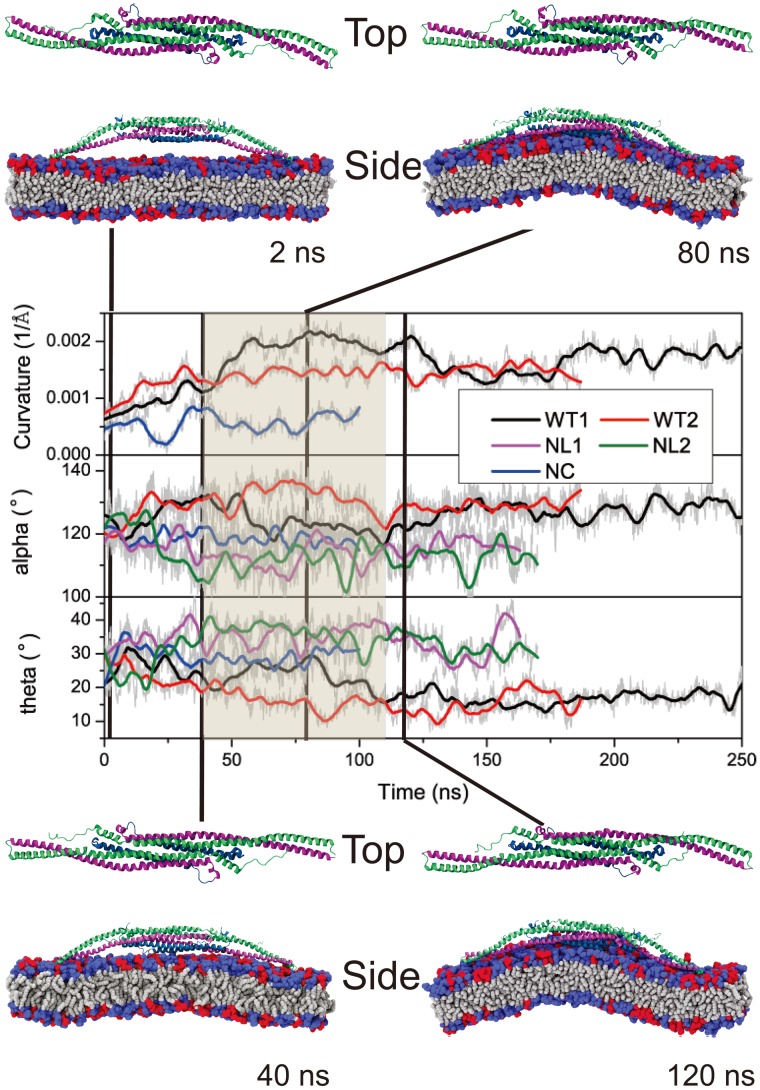
Conformational change of F-BAR domain during interaction with the membrane. Change of membrane curvature and of angles 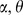 during simulations WT1, WT2, NC, NL1 and NL2 (see Table 1). Original data are shown in gray and running averages over 10 ns in color. Conformations of the F-BAR domain and interaction with the membrane are shown at 0, 40, 80 and 120 ns for simulation WT1. Helices 2 to 4 are colored blue, purple and green, respectively; tails of membrane lipids are colored grey; the neutral DOPC head groups are colored blue and the negatively charged DOPS head groups red.


As shown in Fig. 3, both 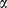 and 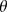 of WT1 change significantly upon interaction with the membrane; 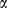 increases up to 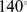, then decreases to 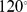, fluctuating finally around 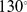; 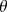 decreases to 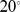, then increases back to 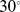, fluctuating finally around 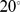. In control systems NL1, NL2 and NC, 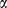 and 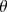 do not show such changes and fluctuate around different average angles.

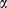
 and 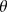 represent the conformational change of the F-BAR domain in the horizontal and vertical direction. A high anti-correlation is found between the change of 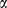 and 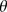 (Pearson correlation coefficient = −0.5), corresponding to a synchronized change of F-BAR domain side helices movement and protein curvature. Visual inspection of the simulation reveals that the anti-correlation of 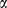 and 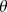 changes correspond to a partial uncoiling movement of the coiled-coil structure formed by side helices 3 and 4 (Fig. 3 and Fig. S6 in Text S1). An increase in 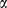 accompanied by a decrease in 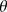 corresponds to the F-BAR domain forming a shallow concave surface; little movement is observed for the central helices (Fig. S4 in Text S1) and all helices retain their helical structures during interaction between the F-BAR domain and the membrane (Fig. S7 in Text S1).

As expected, when the F-BAR domain assumes a concave shape, the attached membrane undergoes induced-fit bending. Unlike N-BAR domains, which act like rigid bodies attracted to a membrane [9], [66], [68], the F-BAR domain and the membrane influence each others shape. Indeed, the bending energy of the F-BAR domain is much lower than that of the N-BAR domain, suggesting that the F-BAR domain is not as rigid as the N-BAR domain [30], [37], [38]. Based on the conformation of the F-BAR domain and membrane curvature, the curvature generation process by the F-BAR domain can be separated into three phases. The curvature generation, in fact, is an induced-fit process, during which membrane binding energy is transfered into membrane bending energy through protein conformational change.

In phase 1, lasting from 0 to 40 ns, the F-BAR domain binds to the membrane and membrane curvature increases slowly, while 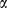 increases and 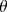 decreases. During this phase, the side helices of the F-BAR domain straighten up and the domain adopts a shallow inner surface, to allow all positively charged residues along the concave surface to contact the negatively charged membrane (Fig. 1C); water molecules between the F-BAR domain and membrane are squeezed out; potential energy is stored in the newly formed F-BAR domain conformation.

In phase 2, lasting from 40 to 120 ns, membrane curvature is generated. During this phase, 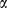 and 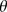 adjust and domain curvature increases. Potential energy stored in the F-BAR domain conformation is released into energy associated with membrane curvature.

In phase 3, lasting from 120 to 250 ns, the protein-membrane system relaxes. Membrane curvature decreases slightly and fluctuates around 0.0015A?^−1^; 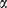 and 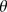 values are restored close to the native state values, indicating partial uncoiling of the coiled-coil structure (Fig. S6 in Text S1). However, 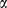 values in simulations NC, NL1 and NL2 are much lower than those in simulation WT1 and WT2, while 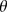 values show the reverse, indicating that the domains coiled-coil structure without interaction with the membrane becomes further coiled, which suggests that partial uncoiling of the domains coiled-coil structure provides the driving force for membrane curvature formation. Indeed, if one removes the membrane from the final conformation of simulation WT1, as is done in simulation WT1WAT, the conformation of the F-BAR domain is quickly restored to a near crystal conformation and 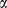 and 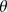 assume values similar to the ones they assume in simulation NL1 and NL2, indicating that the uncoiling is reversible (simulation WT1WAT, see Fig. S8 in Text S1).

The induced-fit interaction between the F-BAR domain and the membrane allows membrane curvature adjustment to F-BAR domain density and lipid type. Indeed, varying F-BAR domain density leads to a wide range of curvatures during membrane structure formation [7], [19], [38], [47], [67]. The F-BAR domain conformational change in response to membrane curvature is likely also a mechanism for membrane curvature sensing. Binding of the F-BAR domain to membranes with curvatures that are significantly different from its intrinsic curvature would require conformational change of the F-BAR domain that is energetically expensive. Indeed, F-BAR domains are found to favor membrane curvatures that match its intrinsic protein curvature [7].

### Theoretical description of the membrane sculpting process

Binding of the F-BAR domain to the membrane leads to a match between shapes of F-BAR domain and membrane. The resulting membrane curvature depends on the balance of two forces, one resisting protein shape changes and the other resisting membrane curvature changes. The bending energy of an F-BAR domain dimer attached to the membrane surface (or any other attached rod-like protein) can be described through [22], [65], [66], [69], [70]

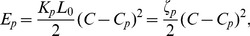
(1)where 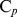 is the intrinsic curvature of the protein, 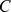 the curvature of the membrane, 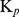 the flexural rigidity of the protein, 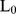 the length of the protein, and 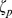 the protein bending rigidity. The curvature-related elastic energy of a cylindrical membrane of length L and radius R can be described by a Helfrich Hamiltonian [71]

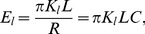
(2)where 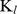 is the membrane bending modulus. The curvature-elastic energy of a membrane patch of length L, width W and radius R can be described analogously by a Helfrich Hamiltonian, namely by
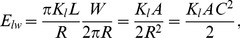
(3)where 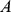 is the area of the membrane patch (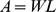).

The total energy of an F-BAR dimer binding to a membrane is then
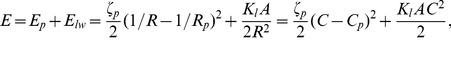
(4)which corresponds to the shape force, 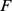,
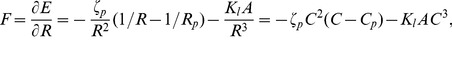
(5)At equilibrium holds F = 0 and, hence,
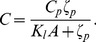
(6)According to the equipartition theorem of thermodynamics holds
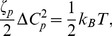
(7)or
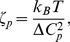
(8)where 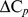 is the curvature fluctuation of the protein, 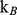 the Boltzmann constant and 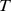 the temperature.

The curvature of the protein was monitored during the last 100 ns of simulation NL1 and is presented in Fig. S9 in Text S1. The intrinsic curvature of the protein was determined as the mean curvature of the protein, namely 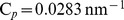, corresponding to a radius of curvature of 35.3 nm. The root-mean square fluctuation of the curvature of the protein was determined from its standard deviation from the average protein curvature and was found to be 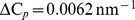. The membrane bending modulus 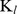 had been measured, through experiments and simulations, to be 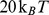 [60], [65], [72]–[75]. According to Eq. 6, the radius of curvature of an F-BAR dimer on top of a lipid patch is then estimated to be 45.1 nm. This value compares well with the radius of curvature monitored during the last 100 ns of simulation WT1, which is 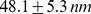.

With the parameters stated above, one can estimate the total binding energy of WT1 F-BAR dimer and membrane patch at equilibrium to be 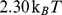, with the bending energy of F-BAR dimer and of membrane patch contributing 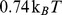 and 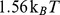, respectively. The average membrane curvature during the early (i.e., phase 1) period 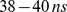 is 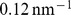 and amounts to the highest membrane curvature during the binding phase. During this period the total energy of the F-BAR-membrane system, the bending energy of the F-BAR dimer and of the membrane patch are 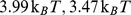 and 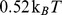, respectively. During the later (i.e., phase 2) period 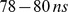 the average membrane curvature is 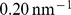 and amounts to the highest membrane curvature during the membrane bending phase. During this period the total energy of the F-BAR-membrane system, the bending energy of the F-BAR dimer and of the membrane patch are 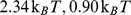 and 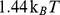, respectively. Therefore, the total energy that is stored in the protein conformational change during membrane binding and membrane bending phases is 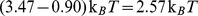. The binding energy can be estimated by the single molecule experiment proposed in [66], in which an F-BAR dimer molecule is pulled away from the membrane at one end.

Binding and close adhesion of the F-BAR domain to the membrane require shape complementarity between protein and membrane. In case that both protein and membrane shapes are radially symmetric, i.e., the centerline of either one obeys in the 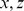-plane the equation 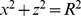, shape complementarity leads to membrane curvature 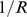. If the F-BAR domains are forming on top of the initially planar membrane a lattice oriented (with the protein major axes) along the 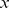-axis then the planar membrane coils into a tube with its long axes pointing along the 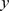-axis.

However, in case that the F-BAR domain does not assume a radial shape, shape complementarity results in an interesting variation. To demonstrate this we assume that the F-BAR domain prefers either intrinsically or through the effect of adhesion to the membrane an ellipsoidal shape governed by the equation 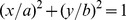 where 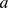 and 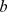 are the major and minor axis of the ellipse. In this case a membrane tube along the 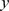-axis does not permit close adhesion as the radially symmetric membrane and the ellipsoidal F-BAR domain don't match exactly. However, a tube tilted by an angle 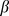 relative to the 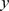-axis permits a perfect match of protein and membrane shape. To see this we note that, according to a well known result of geometry, the tilted tube is cut by the 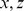-plane along an ellipsoid. One can convince oneself readily that this ellipse has a short axis 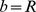 and a long axis 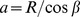. One can then conclude that for the assumed ellipsoidally shaped F-BAR domains (characterized by long axis 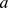 and short axis 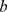), forming a lattice oriented along the 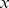-axis on an initially planar membrane, a tube of curvature 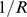 results with direction along an angle 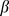 relative to the 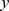-axis, where 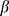 is given by
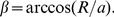
(9)This description assumes binding of the F-BAR domain leading to strong adhesion such that protein and membrane shape match very closely. In any case, a circular membrane tube can accommodate non-circular F-BAR domain shapes by rotating the tube axis, but only shapes that are nearly ellipsoidal. As stated already, such shapes can result from a combination of an intrinsic and an induced shape of the F-BAR domain dimer adhesion surface.

### Membrane curvature generated by F-BAR domain lattices

**Figure 4:**
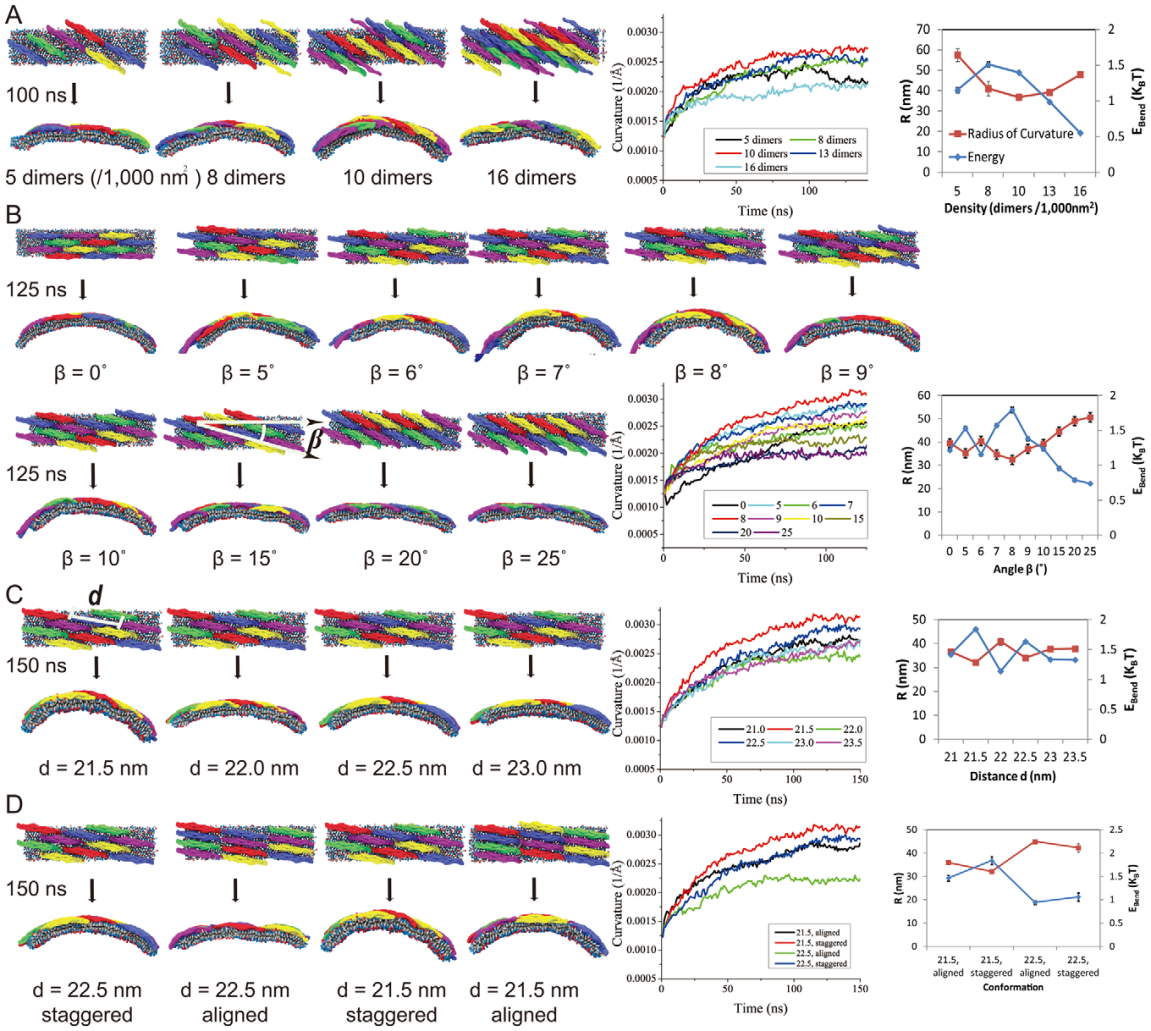
Membrane curvature induced by lattices of F-BAR domains. (A) Dependence of membrane curvature on F-BAR domain density. Shown is curvature generated by lattices with F-BAR dimer densities of 5, 8, 10, 13 and 15 dimers per 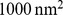. A density of 10 dimers per 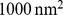 generates the highest curvature, with radius of curvature 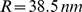. (B) Membrane curvature induced by F-BAR domains forming lattices of different angle 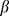. An angle of 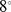 produces the highest curvature, with radius of curvature 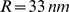. (C) Dependence of membrane curvature on inter-dimer distance. A distance of 21.5 nm produces the highest curvature, with radius of curvature R = 33 nm. (D) Dependence of membrane curvature on staggered or aligned arrangement of F-BAR domains. A staggered arrangement produces higher curvature than an aligned arrangement. F-BAR domains and lipid membranes shown on the left of (A–D) are shown in color and in grey, respectively; individual F-BAR domains are differentiated by color.

As stated already, tubules and liposomes with wide range of curvatures are found to be generated by the F-BAR domain [7], [19], [38], [47], [67]. Apparently, the variation stems from the collective action of the domains as visualized, for example, in cryo-EM images [38]. To investigate how F-BAR domains curve membranes collectively, we built a series of F-BAR domain lattices adopting the SBCG simulation model (see Methods). We performed, for this purpose, four series of simulations with F-BAR domain lattices of varying type. The lattices studied and the resulting curvatures are depicted in Fig. 4.

**Figure 5:**
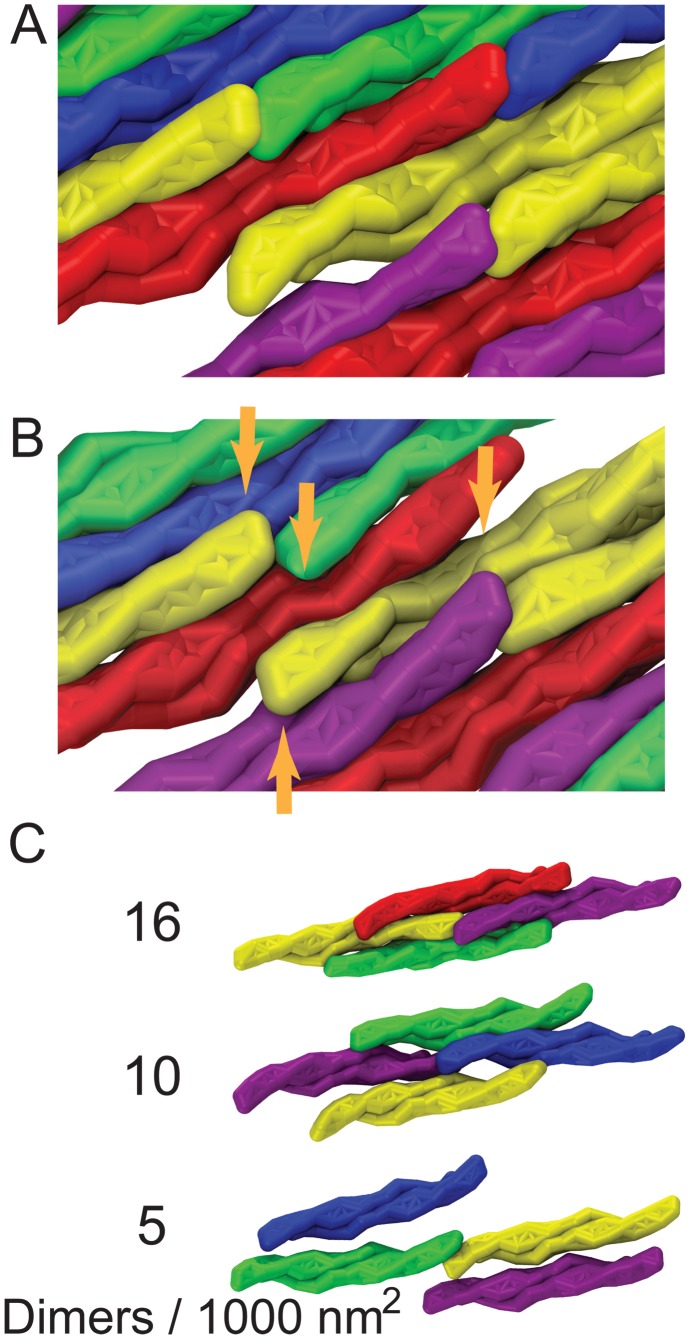
Conformation of F-BAR domain lattices looking up from the membrane towards the protein. Individual F-BAR domains are differentiated by color. Initial (A) and final (B) conformation of the F-BAR domain lattices on top of the membrane taken from one of the systems, simulations LATTICES, shown in Fig. 4; the density is 13 dimers per 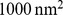. Positions where the concave surface of the F-BAR domains is blocked by neighboring F-BAR domain tips are marked by orange arrows. (C) Parts of F-BAR domain lattices at different densities.

In a series of SBCG simulations, LATTICES (Table 1), we examined how the F-BAR domain density affects membrane curvature. As Fig. 4 shows, of the F-BAR domain lattices with five different densities, the one with 10 dimers per 1000 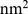 achieves highest curvature; lattices with lower densities achieve much lower curvature. This result is expected since the denser the lattices are, the more the F-BAR domains can act on the same area of lipid. However, membrane curvature becomes also reduced when the F-BAR domain density gets too high, due to neighboring F-BAR domains hindering each others access to the membrane as shown in Fig. 5A. This hinderance of neighboring domains increases as domain density increases (Fig. 5B). The F-BAR domain density generating the narrowest tubules, as seen in cryo-EM [38], is 8 to 10 dimers per 1000 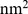.

Fig. 4 shows the relationship between membrane curvature and lattice geometry. Rather diverse curvatures (radii of curvature range from 25 to 100 nm) are seen to be generated by lattices with different parameters [7], [19], [38], [47], [67]. High curvatures are generated by lattices with 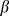 values in the range of 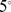
–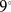. An inter-domain distance of 21.5 nm with the F-BAR domains being staggered in an end-to-shoulder arrangement yields the highest curvature. The results in Fig. 4 are consistent with recent cryo-electron microscopy images of F-BAR domain lattices on membrane tubules [38].

The observed tilt angle 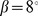 between 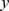-axis and tube axis suggests, according to Eq. 9, that the actual shape of the F-BAR domain membrane adhesion surface is ellipsoidal with axes 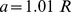 and 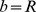, i.e., the widening of the F-BAR domain shape is very small, but significant enough to induce an observable reorientation of the tube axis. To understand how a deviation from circular shape as reflected by 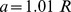 can be significant one should note that the lattice of F-BAR domains averages over the shape effect of many proteins such that even minor effects add up to the tube axis tilt.

### Membrane tubulation by F-BAR domain lattices

**Figure 6:**
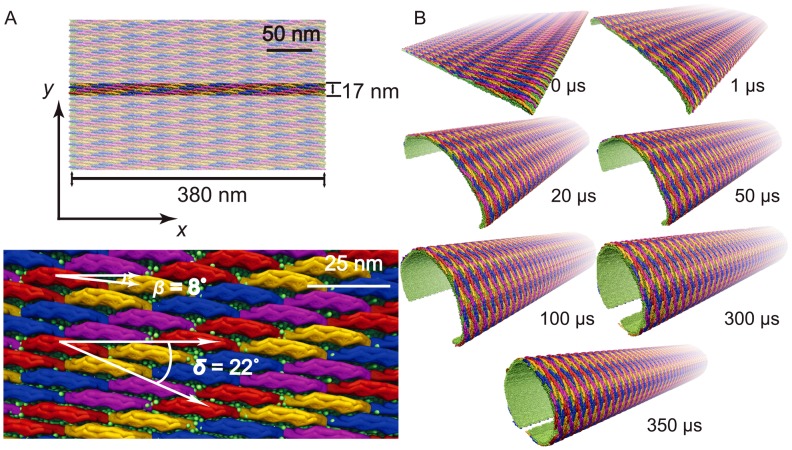
Membrane tubulation by lattices of F-BAR domains. (A) Initial conformation of lattices of F-BAR domains on the membrane in a SBCG representation. A patch of membrane, 17 nm in width, is covered with 4 rows of F-BAR domains, each row containing 17 F-BAR domains. Each individual domain is tilted by 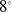 with respect to the 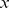-axis and each row of closest contact F-BAR domains is tilted by 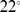 with respect to the 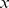-axis. Periodic boundary conditions are assumed in the 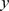-direction, so that the system can be regarded as a lipid patch of infinite length in this direction. (B) Membrane tube formation with lattices of F-BAR domains. Shown are snapshots of membrane structures during the 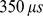 simulation. Membrane lipids are shown in green; individual F-BAR domains are differentiated by color.

**Figure 7:**
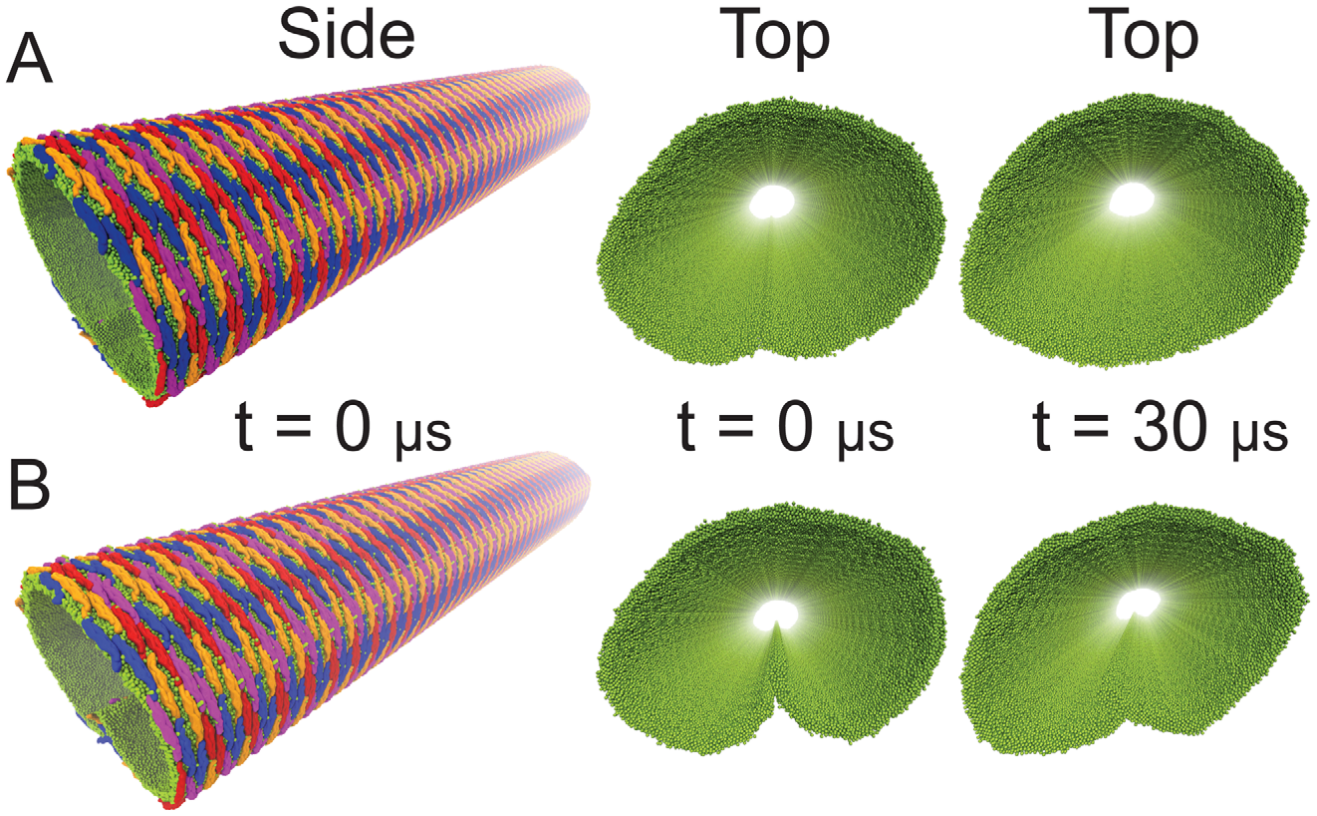
Membrane tubules induced by F-BAR domain lattice. (A) Membrane tubule formed by edge-to-edge fusion. As explained in the text, the 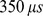 end result of simulation TUBULATION, shown in Figure 6b, had its free edges forcibly fused, the resulting membrane tubule being presented here. The membrane tubule is shown from side and top; in the latter case the F-BAR domains have been removed, leaving solely the lipids, depicted in green; individual F-BAR domains are differentiated by color. Shown is also the membrane tubule structure after 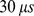 simulation with F-BAR domain removed, quite clearly the tubule structure remained intact. (B) Membrane tubules formed as in (A), but by edge-to-edge fusion forming a T-junction. The membrane tubule is shown from side and top; in the latter case the F-BAR domains have been removed, leaving solely the lipid. Shown is also the membrane tubule structure after 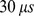 simulation with F-BAR domain removed. Colors are the same as in (A).

To investigate how a complete tubule is formed by a lattice of F-BAR domains, the best (highest curvature induced) performing lattice was placed on a 380 nm wide planar membrane (Fig. 6). Periodic boundary conditions in the 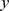-direction imply that the lattice acts on an infinitely long membrane patch. Membrane curvature in simulation TUBULATION (see Table 1 and Methods) developed within hundreds of microseconds from the edges (curving first) to the center (curving last). After 350 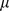
s, a tubular structure with local radius of curvature R = 60–90 nm was formed, with the edges being separated by only 28 nm. In lieu of using more computer time (the simulation stretched over 10 months), we applied a weak radial force until the edges met, fusing the membrane into a complete tubular structure (Fig. 7A). After the tube was closed, we removed all F-BAR domains and carried out 30 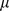
s of further equilibrium simulation, during which the tube remained closed. Tubules formed by the F-BAR domain lattices *in vivo* range from 25 to 100 nm in radius [7], [19], [38], [47], [67]. In a second simulation we observed a tube fusing event in which one edge of a tube met the other edge in a T-like junction. Removing all F-BAR domains and continuing the simulation for 30 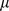
s revealed again a stable structure (Fig. 7B).

**Figure 8:**
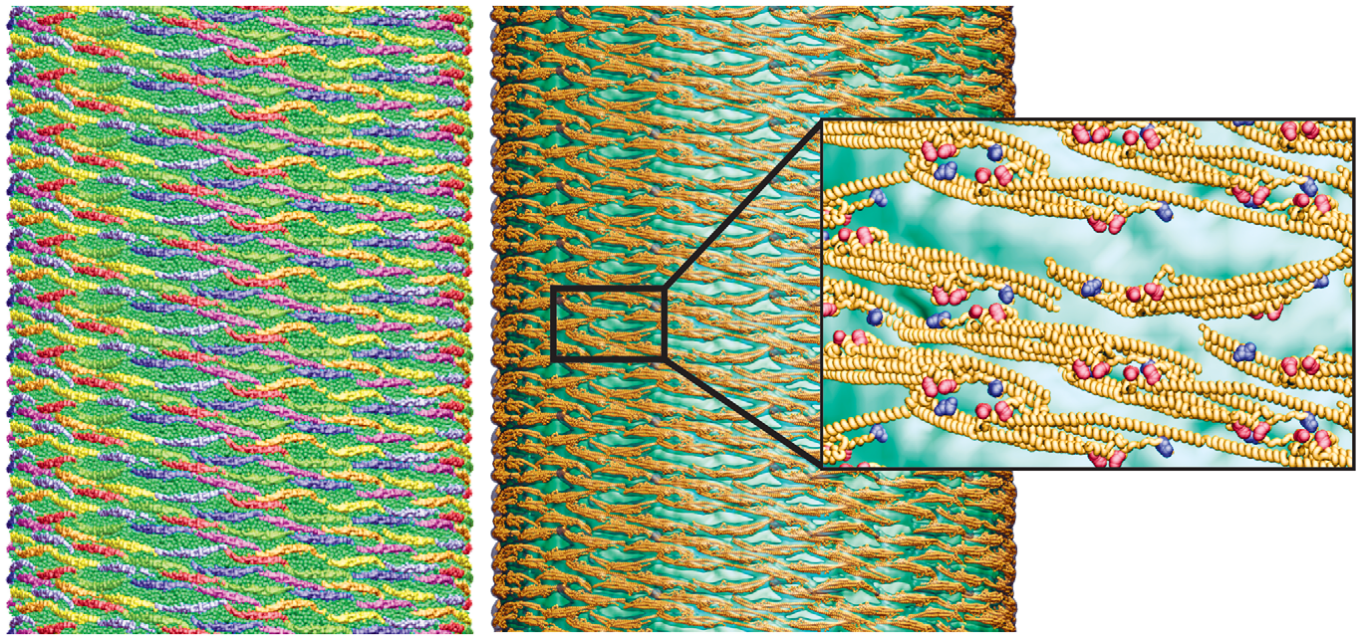
All-atom structure of F-BAR domain lattice on the formed membrane tube. (Left) Coarse-grained tube structure from simulation TUBULATION, as depicted in Fig. 6. (Right) All-atom structure constructed from the SBCG structure. (Insert right) Close up view of all-atom structure, rendered in so-called cartoon representation. Residues Lys66, Arg47 are shown in van der Waals representation and colored in red; Glu285, Asp161 are represented in the same way, but colored in blue.

To study the interactions between F-BAR domains in a tube-forming lattice at all-atom resolution, we aligned all-atom structures of the F-BAR domains with the SBCG model on the surface of the fully formed tubule structure (Fig. 8) employing the method reported in [24]. Analysis of the structure showed that side-to-side contacts are maintained between most pairs of neighboring F-BAR domains, due to a large number of charged residues at the lateral contact points, e.g., Lys66, Asp161 of one dimer and Glu285, Arg47 of another. Indeed, mutation of these residues into neutral amino acids abolishes tubule formation by the F-BAR domains [13], [76], which suggests that the contacts are important for lattice formation and hence, membrane tubulation. Further analysis of the lattice structure revealed that end-to-end contacts are not maintained. This observation is consistent with the cryo-EM images, in which end-to-end contacts are seen not to be strong and are found absent in the narrowest tubule observed [38].

### F-BAR domain lattices allow defects

While most F-BAR domains in our simulations retain their original degree of tilting with 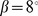, some F-BAR domains exhibit degrees of tilting in the range of 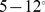. In experiment, F-BAR domain lattices induce membrane tubules not in a manner ordered enough to produce high-quality cryo-EM structures [38]. Instead, cryo-EM structures require several rounds of annealing. Therefore, it is likely that in cells F-BAR domains form lattices considerably more random than seen in cryo-EM. Indeed, N-BAR protein coats on tubule surfaces are found to be dynamic and with a tendency to scramble [21], [38].

Additionally, one out of the 167 F-BAR domains was found in our simulation to assume a side-laying orientation, where the F-BAR domain turns 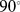 around its principal axis. In the side-laying orientation the F-BAR domain forms membrane contact with its side surface, rather than the concave surface, and the side-to-side contacts between neighboring F-BAR domains are abolished. The side-laying orientation is only observed at large defects of the F-BAR domain lattices, where local F-BAR domain concentration is low. The side-laying state has also been observed in the all-atom simulations WT1DEL (Table 1). Indeed, in experiment the side-laying state has been observed to induce tubules with low curvatures and at low BAR domain density [9], [38]. It is likely that in cells, both the upright and the side-laying orientation arise in the F-BAR domain lattice. Both side-to-side contacts between the F-BAR domains and the short loop of residues 56 to 60 are important in maintaining orientation in the F-BAR domain lattices.

### Conclusions

In summary, our study on membrane sculpting by F-BAR domains resolves in atomic detail how F-BAR domains sculpt curved membranes. All-atom MD simulations show F-BAR domains dynamically interacting with a membrane, revealing that F-BAR domains sculpt membranes according to the scaffolding mechanism. F-BAR domains act in three steps, namely binding to the membrane, bending the membrane and equilibration. Positively charged residues along the concave surface of the F-BAR domain play a key role in attracting negatively charged membrane lipids towards the F-BAR domain concave side, though F-BAR domains do not act as rigid templates.

We also performed a 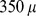
s CG simulation providing a detailed, dynamic picture of membrane tubulation by an F-BAR domain lattice. Depending on the F-BAR domain arrangement within lattices, a wide range of membrane curvatures can be generated. Lattices that generate the greatest curvature (radius of curvature R = 28 nm) involve an F-BAR domain density of 8 to 13 dimers per 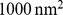, a tilting angle 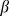 of 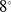, an inter-dimer distance of 21.5 nm and end-to-shoulder contacts. Both side-to-side contacts between F-BAR domains and, in particular, a short loop of residues 56 to 60 are important in maintaining the F-BAR domain in the upright conformation. Our approach combined all-atom and SBCG simulations and revealed how strikingly beautiful F-BAR domain lattices generate large scale membrane shapes in living cells.

## Methods

**Figure 9:**
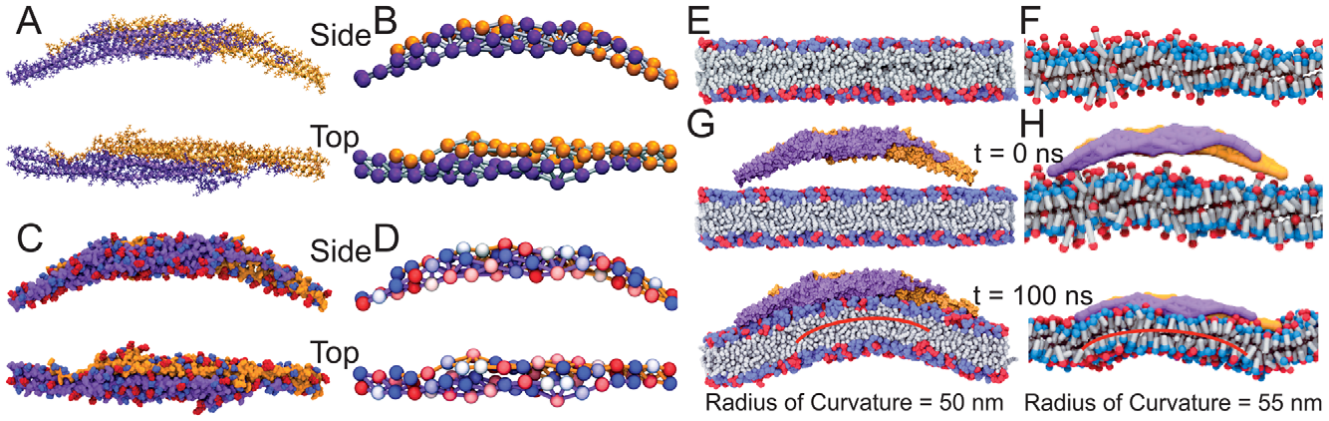
All-atom and SBCG model of F-BAR domain and membrane. The F-BAR domain is shown as the all-atom (A) and the SBCG (B) model in side-view and top-view, with monomers differentiated by colors purple and orange. Charge distribution of all-atom (C) and SBCG (D) F-BAR domain. In the all-atom model, positively and negatively charged residues are shown in red and blue, respectively. In the SBCG model, the charge on each bead is color-coded on a scale from 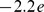 (blue) to 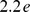 (red). All-atom (E) and SBCG (F) model of the DOPC/DOPS membrane. The neutral DOPC head groups are colored blue, and the negatively charged head groups on DOPS are colored in red. Starting (top) and final (bottom) conformation of all-atom (G) and SBCG (H) model of a single F-BAR domain and membrane.

The atomic coordinates of Homo sapiens EFC/F-BAR domain were taken from Protein Data Bank (pdb code: 2EFK) [13]. Nine residues missing at the N-terminus of EFC/F-BAR domain were modeled based on residue 1 to 9 present in the highly homologous FBP17/F-BAR domain (pdb code: 2EFL) [13]. In all simulations reported here an F-BAR domain homo-dimer was employed as a protein unit, since the homodimer is expected to be the active form of the protein as shown in experiments [7], [13], [38]. The dimer conformation is shown in Fig. 9A. Lipid membranes composed of 67% dieleoylphosphatidylcholine (DOPC) lipids (neutral) and 33% dioleoylphosphatidylserine (DOPS) lipids (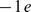 charged) were assumed in all simulations; the latter were performed with NAMD 2.7 [77].

### All-atom simulations

All-atom simulations were carried out to study membrane bending by a single F-BAR domain. For simulations WT1, WT2 and NC, a flat lipid membrane of 150 DOPC molecules was constructed with the VMD membrane builder tool [78]. After 10 ns of equilibration, 33% of lipid headgroups were randomly chosen to be mutated to DOPS headgroups. The DOPC/DOPS membrane was then equilibrated for 20 ns. After replicating the DOPC/DOPS membrane to reach a dimension of 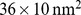, an F-BAR domain dimer was placed on top of the resulting patch with no initial contacts to the membrane. For all all-atom simulations, sodium and chloride ions were added to neutralize the simulated systems and to reach an ion concentration of 0.15 M/L; the TIP3P water model [79] was used for solvation. The resulting models contained 0.4 M atoms. The systems in simulation WT1, WT2 and NC were equilibrated for 1 ns with protein and lipid atoms constrained to their initial positions (with spring constant 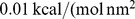)) and then simulated without any constraints for 250 ns, 175 ns and 80 ns, respectively.

In simulation WT1WAT, the final conformation, i.e., the one at 250 ns, was taken from simulation WT1 and the membrane removed. The system was then solvated and equilibrated with the same procedure as WT1. In simulation WT1DEL, the final conformation was taken from simulation WT1 and residues 56 to 60 of the protein removed. The system was then solvated and equilibrated using the same procedure as for WT1. In simulation NC, positive charges along the concave surface of the F-BAR domain were abolished on the following residues: Lys171, Lys173, Lys27, Lys30, Lys33, Lys110, Arg113, Lys114, Arg121, Arg122, Lys132, Lys138, Arg139, Lys140, Arg146, Lys150, Lys51, Lys52, Arg57; this was achieved by adding −0.25 charge to NZ, HZ1, HZ2, HZ3 atoms of lysine residues and −0.5 charge to NH1 and NH2 atoms of arginine residues, while preserving the protein structure.

For the all-atom simulations the CHARMM27 force field with CMAP correction for proteins and ions [80], [81], alongside TIP3P water [79], was assumed. Simulation details can be found in [24], [60]. The simulations described an NPT ensemble; temperature was maintained at 310 K through a Langevin thermostat with a damping coefficient 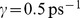 [82]; pressure was maintained at 1 atm with a Langevin-piston barostat [82]. Short-range non-bonded interactions were cut off smoothly between 1 and 1.2 nm; long-range electrostatics was computed with the PME algorithm [83]; simulations were performed with an integration time step of 2 fs in NAMD 2.7 [77].

### Shape-based coarse-grained simulations

The shape-based coarse-grained (SBCG) method was developed to simulate protein and lipid assemblies and had been applied successfully to study viral capsids [60], [84] as well as N-BAR domain protein [24], [25], [60]. For the current study the F-BAR domain protein was represented by 60 CG beads arranged according to the protein's shape, corresponding to 150 atoms per bead. The conformation of the SBCG F-BAR dimer matches closely that of the all-atom F-BAR domain (Fig. 9B).

Mass and charge of individual CG beads were based on total mass and charge of the corresponding part of the all-atom protein, resulting in similar charge distributions of all-atom and SBCG F-BAR domain (Fig. 9C, D). Protein shape is maintained in SBCG simulations by harmonic bond and angle potentials 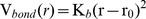 and 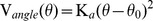, respectively, obtained from all-atom simulations as described in [24]. Initial estimates of force field parameters 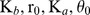 were obtained in the present study through Boltzmann inversion (Fig. S10 in Text S1). To match 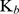 and 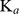 to all-atom simulations, an iterative approach was employed. This approach was automated using the following equations.
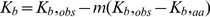
(10)

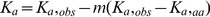
(11)

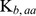
 and 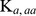 are bond and angle constants obtained from the all-atom simulation, and 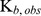 and 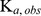 are bond and angle constants obtained from coarse-grained simulations from each iterative refinement; 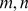 are constants. 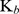 and 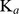 obtained from the formula above was adopted iteratively in a series of SBCG simulations for further and eventually converged refinement. After several rounds of testing, 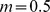 and 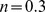 were established as giving bond and angle parameters that best agreed with all-atom simulations and were adopted for iterative refinement (Fig. S10 in Text S1). Pearsons correlation coefficients between the parameters obtained from SBCG and all-atom simulations were 0.965 for 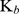 and 0.964 for 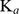. As in [60], each SBCG lipid is composed of a head bead and a tail bead, connected by a harmonic bond. Each bead represents 2.2 DOPC or DOPS lipid molecules on average. The conformation of the all-atom and SBCG lipids is shown in Fig. 9E, F.

A Langevin equation was used to simulate the CG beads as described in [24], with solvent being modeled implicitly. A uniform dielectric constant 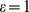 was assumed as rationalized in [24]. Simulations of F-BAR domains on top of a membrane patch in both all-atom and SBCG representations indicated that SBCG matches the time-scale and the membrane curvature of all-atom simulations quite well (Fig. 9G, H and Fig. S11 in Text S1). However we did not observe, in case of the coarse-grained model, the binding-bending phases as seen in the all-atom simulations. Since the binding-bending phases occur on a nanometer scale, while the coarse-grained model (150 atoms per bead) resolves only a 10-to-50 nm scale, the latter model cannot reproduce the detailed energy landscape of the all-atom model. However, the coarse-grained model was calibrated based on the all-atom forcefield and, therefore, matches overall properties of the all-atom model (Fig. 9 and Fig. S10 in Text S1). The main objective of the coarse-grained simulations carried out is to study, on a 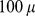
s timescale and on a 100 nm length scale, the collective action of F-BAR domains forming lattices.

All SBCG simulations were carried out with NAMD 2.7 [77]. The integration time step was 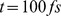. Periodic boundary conditions were assumed. In the longer dimension of the periodic cell, i.e., along the 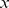-axis, the membrane was discontinuous (with free edges) to permit membrane bending and tube formation. A periodic box of dimension 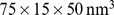 was used for simulations LATTICES and one of dimension 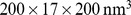 for simulation TUBULATION (Table 1). The simulations described an NVT ensemble; a Langevin thermostat was used to maintain temperature at 310 K [82]. The membrane was a randomized mixture of 67% neutral and 33% negatively-charged lipids, i.e., the same as in the all-atom simulations. In simulation TUBULATION (Table 1), the membrane patches were 380 nm in length, corresponding approximately to the circumference of a tube of 60 nm radius; the simulation was carried out for 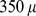
s. The conformation reached at 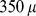
s (see Fig. 6b) was taken and, in order to make membrane edges meet and fuse together, a force of 0.67 pN was applied to the F-BAR domain atoms towards the center of mass of the system using the gridforce method [85]. At the beginning of simulations LATTICES and TUBULATION, F-BAR domain dimers were placed in a regular arrangement (lattice) on top of the DOPC/DOPS membrane without initial contacts to the membrane.

### Visualization and analysis

Analysis and visualization were performed using VMD [78]. As in [24], the tail bead positions of the SBCG lipids define an 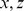-plane, with the 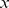-axis being defined by the longest dimension of the unit cell membrane patch at time 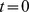 and the 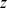-axis being perpendicular to the membrane patch at time 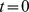. The radius of curvature of the membrane was calculated by least-squared fitting of a circle to the obtained membrane profile in the 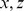-plane. No significant membrane curvature developed in the 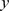-direction. Sequence and structural conservation analysis was performed with the multiseq plugin of VMD [86]; secondary structure analysis of F-BAR domains was performed using the timeline plugin of VMD [78].

## Supporting Information

Text S1 Supplementary Figures S1–S11 on structural features of the F-BAR domain, on the behavior of key residues and on simulation parameters.
(PDF)

Video S1 Video of simulation WT1 trajectory, corresponding to Fig. 1.
(WMV)

Video S2 Video of simulation NC trajectory, corresponding to Fig. 1.
(WMV)

Video S3 Video of simulation TUBULATION trajectory, corresponding to Figs. 6 and 7.
(WMV)
