# Supplementary material for: Membrane Sculpting by F-BAR Domains Studied by Molecular Dynamics Simulations
Source: PLoS Comput Biol. 2013 Jan 31;9(1):e1002892. doi: 10.1371/journal.pcbi.1002892 (PMC3561051; doi:10.1371/journal.pcbi.1002892)
Supplement: Text S1 — Supplementary Figures S1–S11 on structural features of the F-BAR domain, on the behavior of key residues and on simulation parameters. (PDF) [file pcbi.1002892.s001.pdf]

Membrane Sculpting by F-BAR Domains Studied by Molecular Dynamics Simulations

Hang Yu[1,2], Klaus Schulten[1,2,3,*]
1 Beckman Institute, University of Illinois, Urbana, Illinois, USA
2 Center of Biophysics and Computational Biology, University of Illinois, Urbana, Illinois, USA
3 Department of Physics, University of Illinois, Urbana, Illinois, USA
∗ **E-mail: kschulte@ks.uiuc.edu**

Supplementary Figures S1 - S11

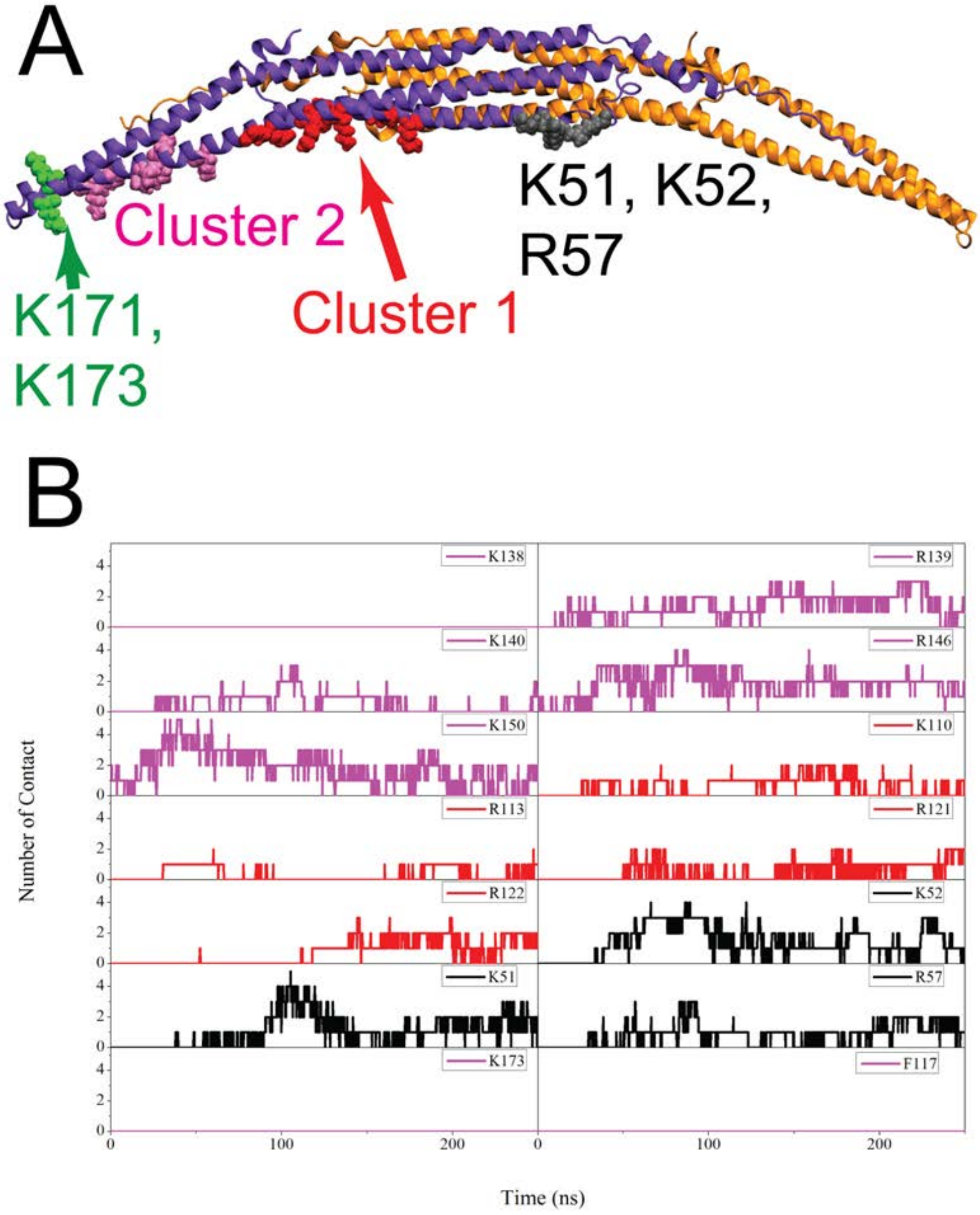

Figure S1. Location of key positively charged residues on the F-BAR domain and their interaction with the membrane in simulation WT1. (A) F-BAR proteins are colored in blue and orange to distinguish the monomers. Colored red is cluster 1, which includes residues Lys27, Lys30, Lys33, Lys110, Arg113, Lys114, Arg121 and Arg122. Colored purple is cluster 2, which includes residues Lys132, Lys138, Arg139, Lys140, Arg146 and Lys150. Colored black are positively charged residues near the short loop of residues 56 to 60, which include residues Lys51, Lys52 and Arg 57. Colored green are positively charged residues at the end of the helix, which include residues Lys171 and Lys173. Residues Lys138, Lys173 and Phe117 do not form contact with negatively charged lipids. (B) Number of contacts formed between negatively-charged DOPS lipid headgroups and positively charged residues along the inner surface of F-BAR domains. A contact is considered formed if nitrogen atoms of Arg/Lys residues are within 5 Å of an oxygen atom of a DOPS lipid headgroup. Contacts of representative residues with lipid are colored in purple, red and black, the colors corresponding to the clusters defined in (A).

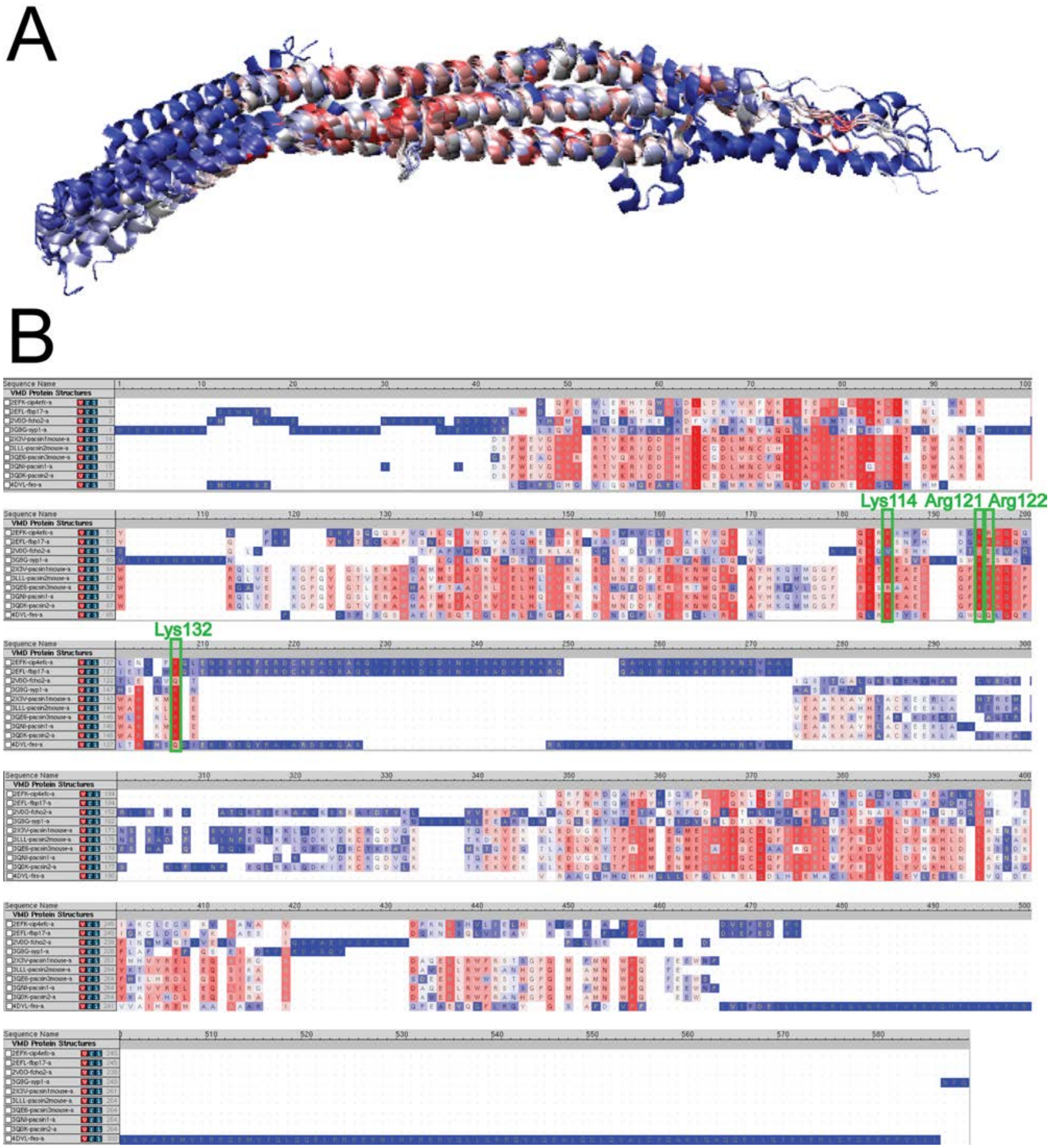

Figure S2. Sequence conservation of F-BAR domain family proteins. (A) F-BAR domain family protein monomer colored according to sequence conservation; conserved residues are colored red, and non-conserved residues are colored blue. (B) Alignment of F-BAR domain family protein monomers colored according to sequence conservation. Key positively charged residues are highlighted in green.

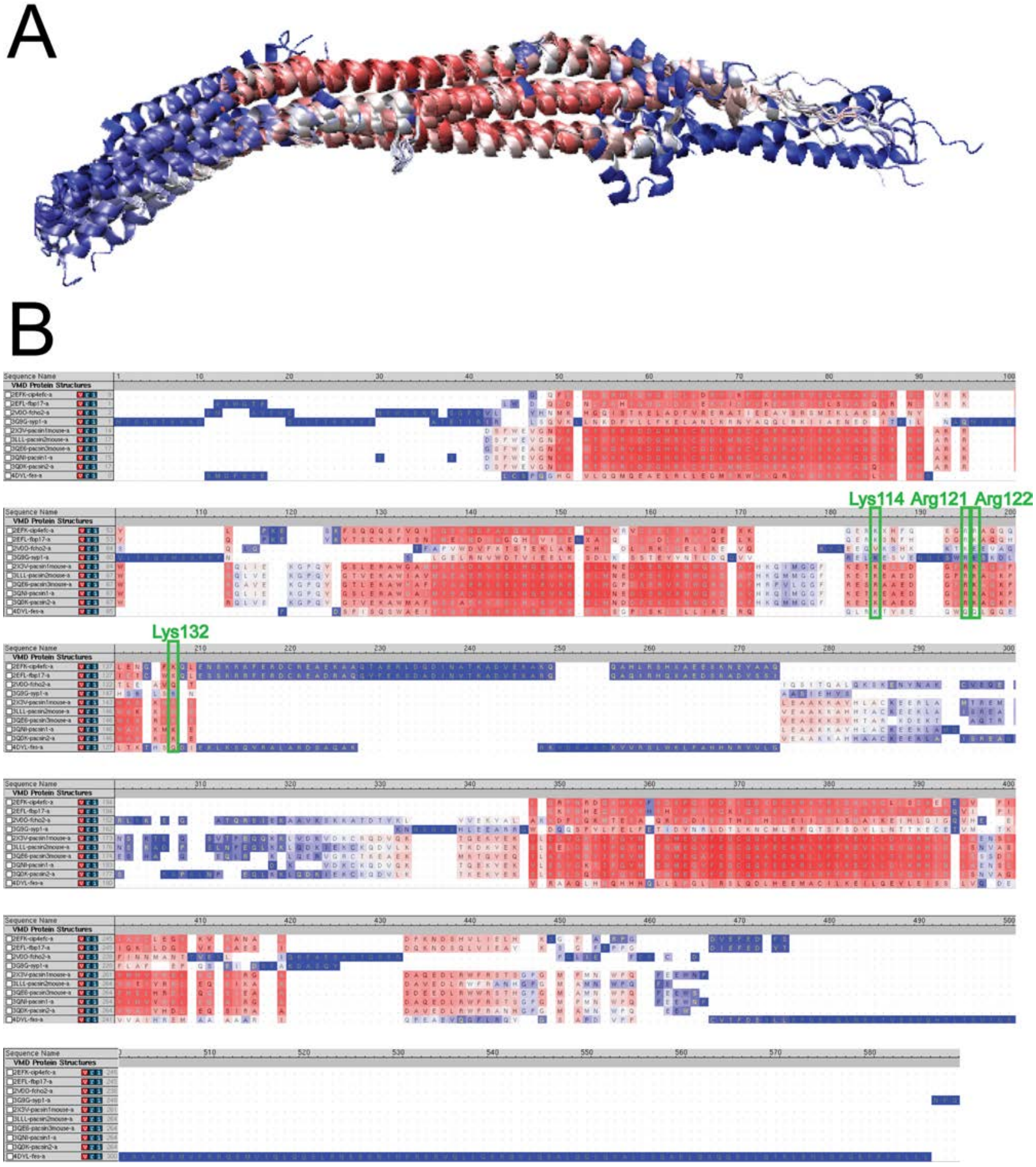

Figure S3. Structural conservation in F-BAR domain family proteins. (A) F-BAR domain family protein monomer colored according to structural conservation; conserved residues are colored red, and non-conserved residues are colored blue. (B) Alignment of F-BAR domain family protein monomer colored according to structural conservation. Key positively charged residues are highlighted in green.

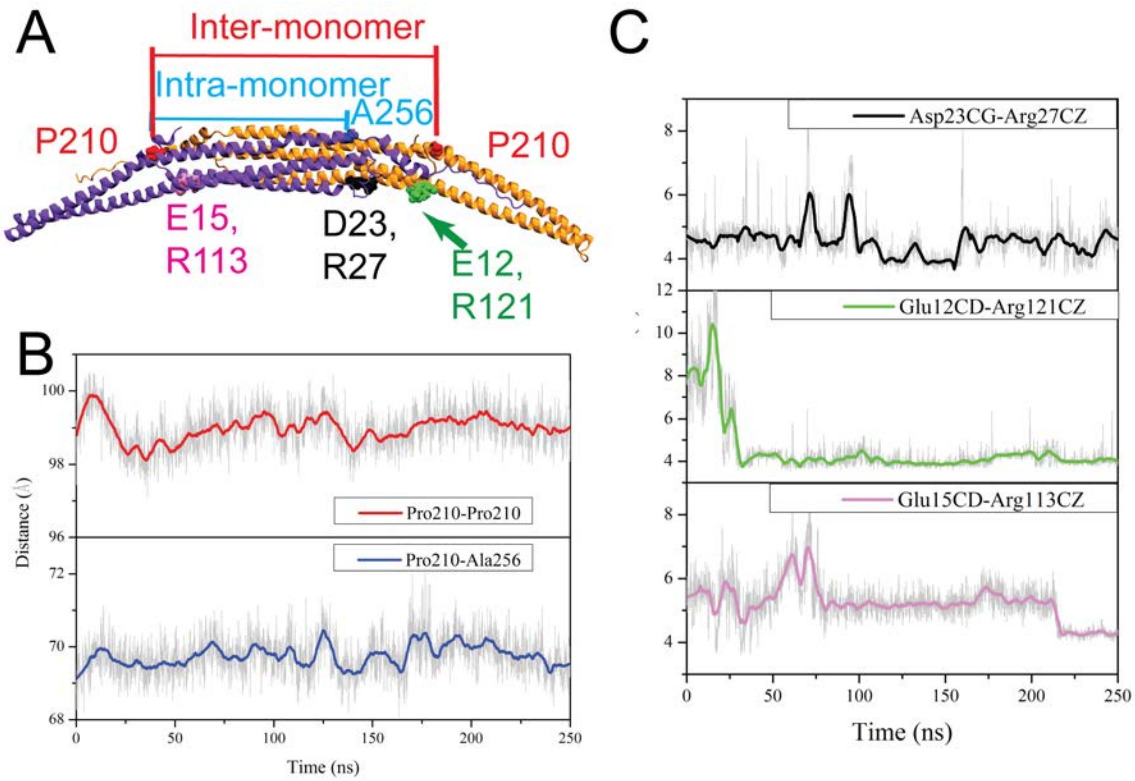

Figure S4. Behavior of key residues in F-BAR domain simulation WT1. (A) Location of the key residues on the F-BAR domain. F-BAR proteins are colored in blue and orange to distinguish the monomers. Colored red are the Pro210 residues of the two monomers; the pro210-pro210 distance reflects inter-monomer motion. Colored blue is residue Ala256; the Pro210-Ala256 distance characterizes intra-monomer motion. Three internal salt bridges are formed by residues Glu15 and Arg113, Asp23 and Arg27, Glu12 and Arg121 colored purple, black and green, respectively.
(B) Inter-monomer and intra-monomer movement of F-BAR domain dimer. Colored red is the distance between C_α atom of Pro210 on two monomers, the distance characterizing inter-monomer motion. Colored blue is the distance between C_α atom of Pro210 and of Ala256, the distance characterizing intra-monomer motion. Original data are shown in gray and running averages over 10 ns in color. Interactions between F-BAR domain and membrane do not induce conformational change in the central helices. (C) Dynamics of three internal salt bridges characterized through the respective bond distances. Colored purple is the distance between CZ atoms of Arg27 and CG atom of Asp23; colored black is the distance between CZ atoms of Arg121 and CD atom of Glu12; colored green is the distance between CZ atoms of Arg113 and CD atom of Glu15. Original data are shown in gray and running averages over 10 ns in color. Arginine residues 27, 121, 113 interact with neighboring residues and at the same time with membrane lipids. See also Fig. 1 and Fig. S1.

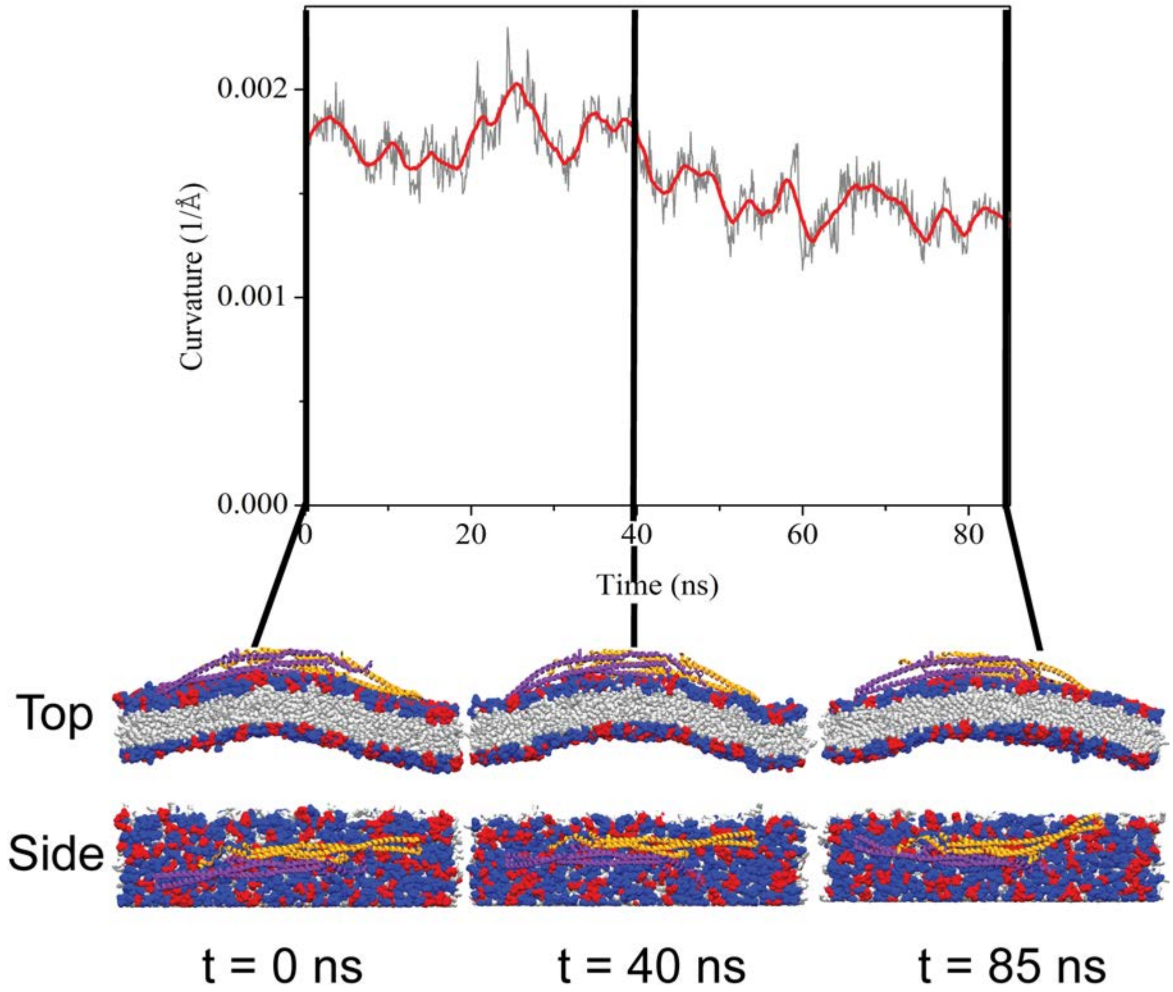

Figure S5. Change of curvature during simulation WT1DEL (see Table 1). Original data are shown in gray and running averages over 5 ns in red. Snapshots of the F-BAR domain-membrane system are shown at 0, 40 and 85 ns. F-BAR proteins are colored in blue and orange to distinguish the monomers. Tails of membrane lipids are colored grey; the neutral DOPC head groups are colored blue and the negatively charged DOPS head groups red. The membrane curvature does not change until 40 ns, and from then on the F-BAR domain turns towards one side, assuming finally a side laying state.

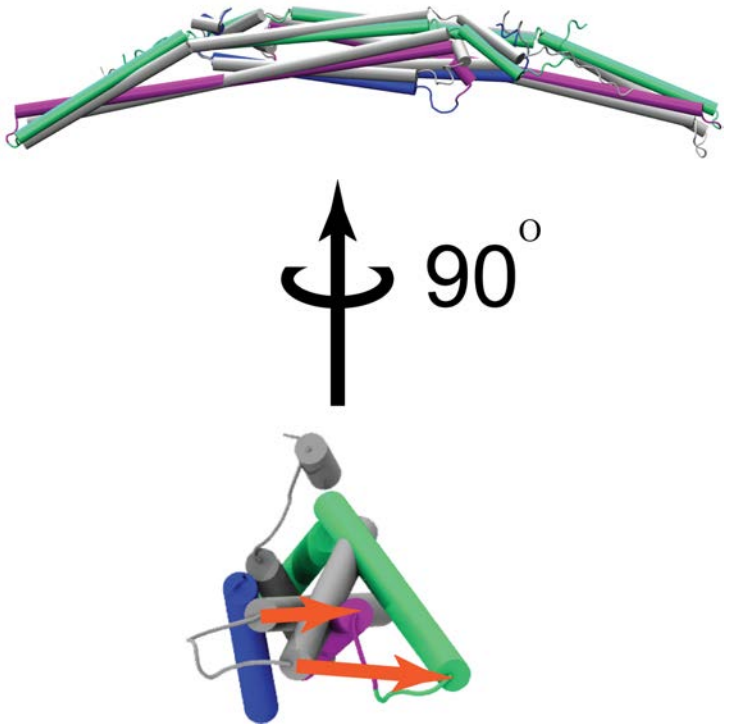

Figure S6. Coiled-coil motion of F-BAR domain side helices during the first 40 ns of simulation WT1. The F-BAR domain at 0 ns is shown in grey and at 40 ns in color. Helices 2, 3 and 4 at 40 ns are shown in blue, purple and green, respectively. Red arrows indicate the movement of helices 3 and 4. Interactions between F-BAR domain and membrane induce partial uncoiling of the coiled-coil structure of the F-BAR domain.

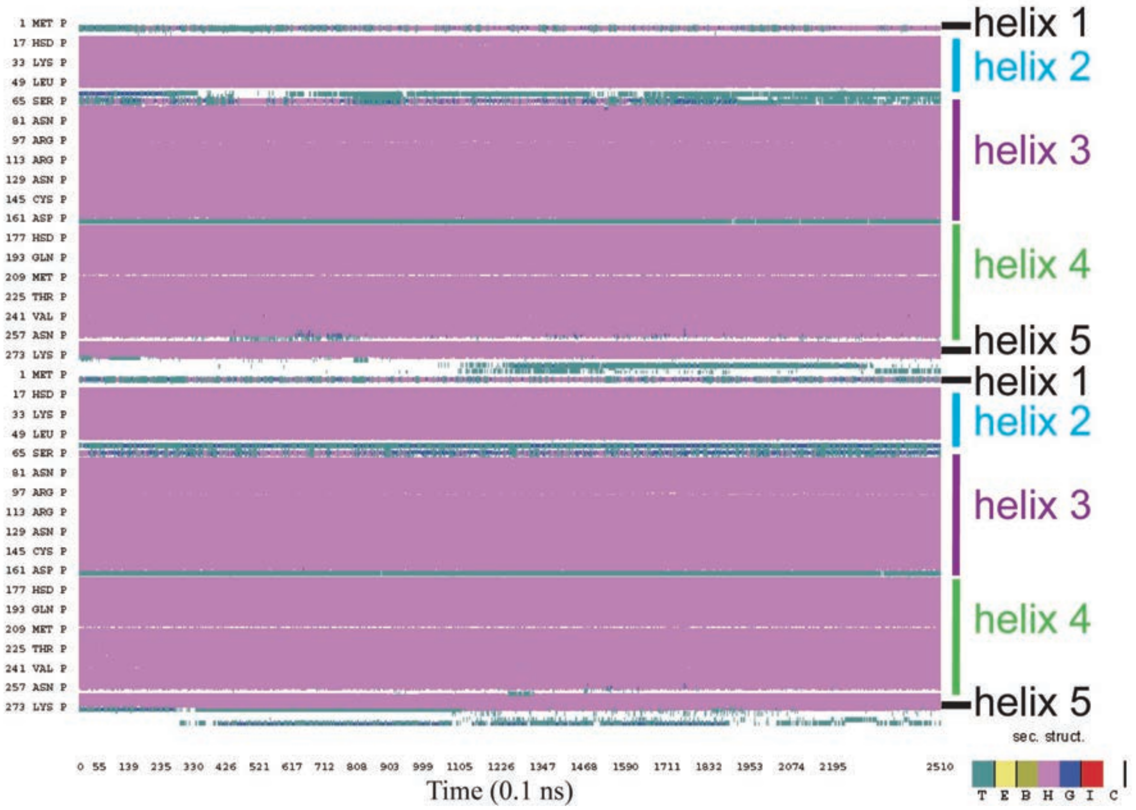

Figure S7. Secondary structure evolution of the F-BAR domain during simulation WT1. Turn (T), extended conformation (E), isolated bridge (B), alpha helix (H), 3-10 helix (G), pi-helix (I) and coil (C) structures are colored in green, yellow, brown, purple, blue, orange and white, respectively. The five helices of each F-BAR monomer are shown in order 1, 2, 3, 4, 5 in black, blue, purple, green and black, respectively. Interactions between F-BAR domain and membrane do not induce helical structure change.

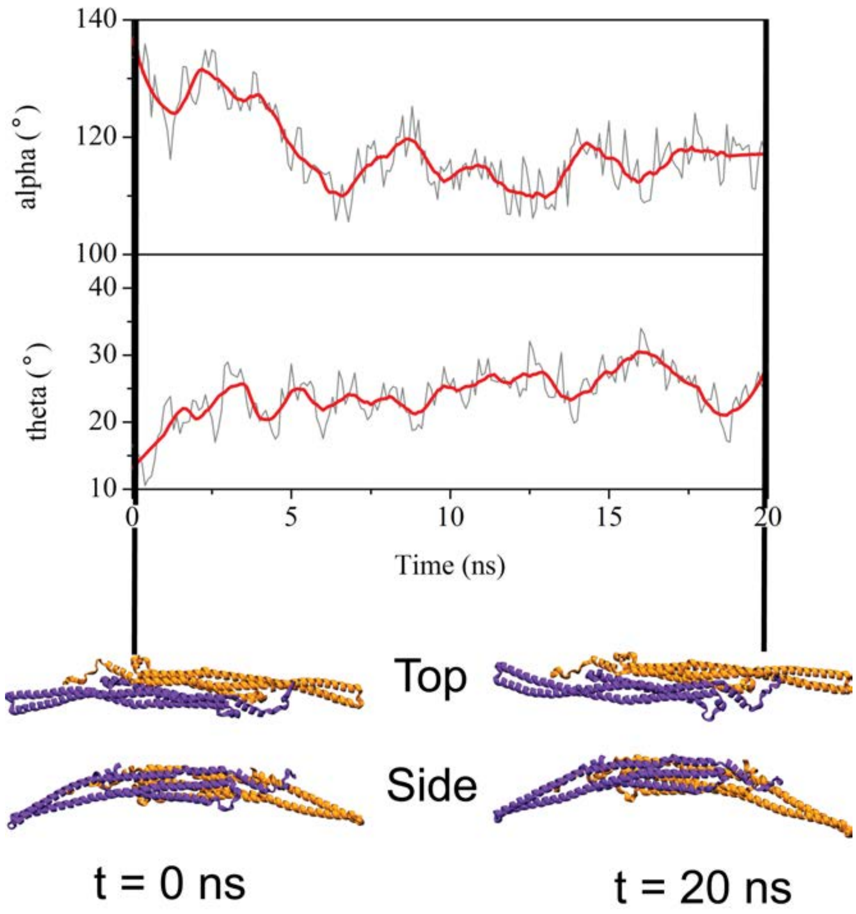

Figure S8. Change of angles α, θ during simulation WT1WAT (see Table 1). Original data are shown in gray and running averages over 2 ns in red. Snapshots of the F-BAR domain are shown at 0 and 20 ns for simulation WT1WAT. F-BAR proteins are colored in blue and orange to distinguish the monomers. Simulation WAT1WAT described the F-BAR domain dimer in water, without lipid membrane, the membrane having been removed from the final conformation of simulation WT1 (see Methods). Within 5 ns, both angle α and θ return to values similar to those seen in simulation NL1 and NL2, and the curvature of the F-BAR domain as well as the tilting angles of the side helices are restored close to the native state values.

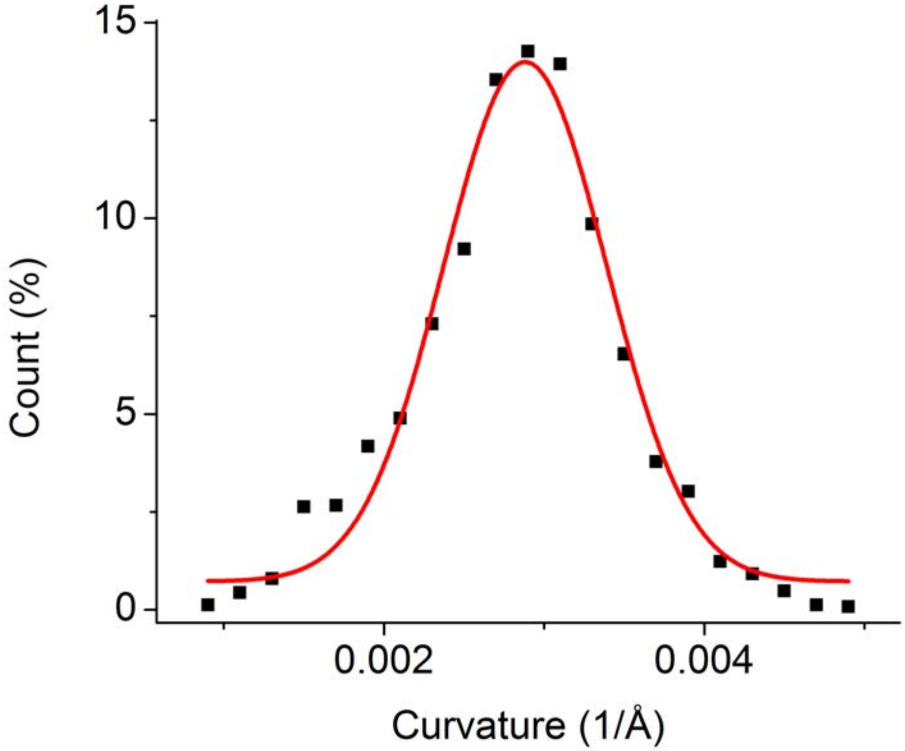

Figure S9. Curvature distribution of simulated F-BAR domain. Protein curvature from the last 100 ns of simulation NL1 are shown as black dots. A Gaussian fit to the distribution is shown in red (average = 0.0029 1/Å, halfwidth = 0.001 1/Å).

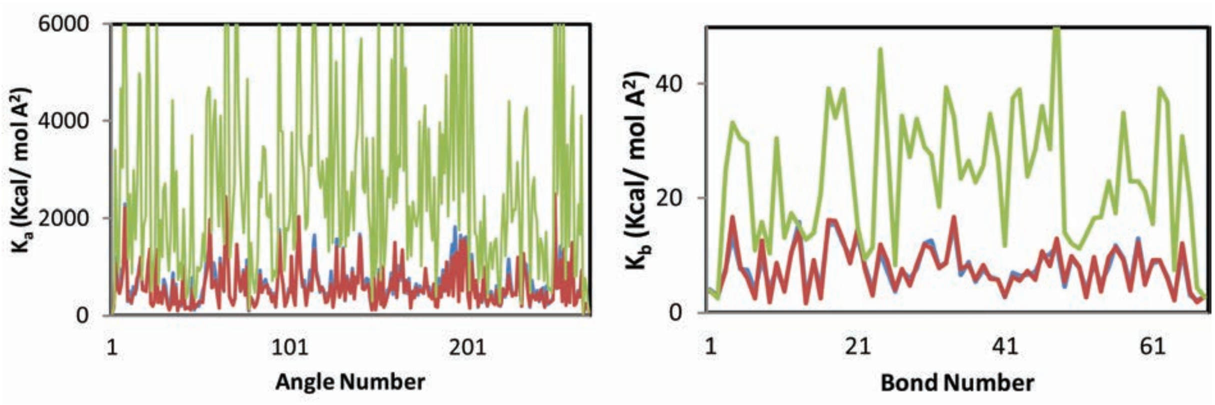

Figure S10. Bond and angle parameters of the SBCG simulation in comparison with those of the all-atom simulation. Bond and angle parameters obtained from Boltzmann inversion of the all-atom simulation are shown as a green line, those from the all-atom simulation as a blue line and those from automated iterative refinement as a red line.

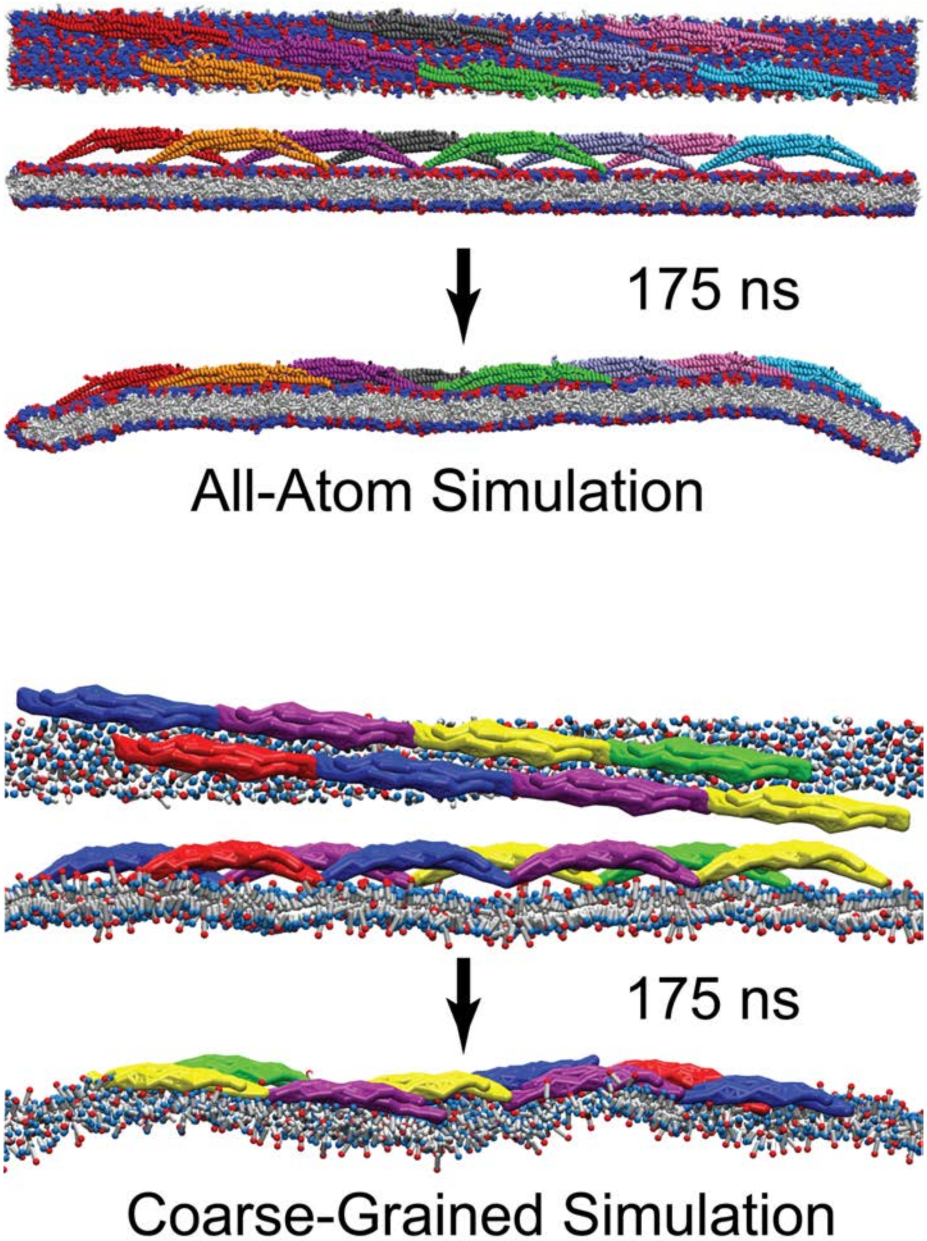

Figure S11. Comparison of simulations 8-F-BARs (all-atom) and SBCG 8-F-BARs (SBCG, see Table 1). Conformation of 8 F-BAR domains on top of a lipid patch are shown at t = 0 and t = 175 ns. Individual F-BAR domains are differentiated by color; tails of membrane lipids are colored grey; the neutral DOPC head groups are colored blue and the negatively charged DOPS head groups red.
